# A missing jigsaw within the hygiene hypothesis: Low-dose bisphenol A exposure attenuates lipopolysaccharide-induced asthma protection

**DOI:** 10.1093/pnasnexus/pgad312

**Published:** 2023-11-07

**Authors:** Mengjing Wang, Jing Qu, Junjie Yang, Tian Zhang, Wei Ren Tan, Shumin Liao, Xing Chen, Yingzi Liu, Xiang Long, Xue Li, Yun Xia, Nguan Soon Tan, Liang Li, Mingliang Fang

**Affiliations:** Department of Environmental Science and Engineering, Fudan University, Shanghai 200433, China; School of Civil and Environmental Engineering, Nanyang Technological University, Singapore 639798, Singapore; Department of Pathogen Biology, Shenzhen Center for Disease Control and Prevention, Shenzhen 518055, China; School of Civil and Environmental Engineering, Nanyang Technological University, Singapore 639798, Singapore; Lee Kong Chian School of Medicine, Nanyang Technological University, Singapore 639798, Singapore; Lee Kong Chian School of Medicine, Nanyang Technological University, Singapore 639798, Singapore; Department of Pharmacology, School of Medicine, Southern University of Science and Technology, Shenzhen 518055, China; Institute of Mass Spectrometer and Atmospheric Environment, Jinan University, Guangzhou 510632, China; Intervention and Cell Therapy Center, Peking University Shenzhen Hospital, Shenzhen 518036, China; Department of Respiratory Medicine and Critical Care, Peking University Shenzhen Hospital, Shenzhen 518036, China; Institute of Mass Spectrometer and Atmospheric Environment, Jinan University, Guangzhou 510632, China; Lee Kong Chian School of Medicine, Nanyang Technological University, Singapore 639798, Singapore; Lee Kong Chian School of Medicine, Nanyang Technological University, Singapore 639798, Singapore; Department of Pharmacology, School of Medicine, Southern University of Science and Technology, Shenzhen 518055, China; Shenzhen Institutes of Advanced Technology, Chinese Academy of Sciences, Shenzhen 518055, China; Joint Laboratory of Guangdong-Hong Kong Universities for Vascular Homeostasis and Diseases, Shenzhen 518055, China; Department of Environmental Science and Engineering, Fudan University, Shanghai 200433, China

**Keywords:** allergic asthma, environmental pollutants, lipopolysaccharides (LPSs), bisphenol A (BPA), house dust mite (HDM), asthma pathophysiology

## Abstract

The rising occurrence of allergic asthma in early life across industrialized countries suggests that environmental factors play a crucial role in determining asthma susceptibility and severity. While prior exposure to microbial lipopolysaccharides (LPSs) has been found to offer protection against allergic asthma, infants residing in urban environments are increasingly exposed to environmental pollutants. Utilizing limulus lysate test screens and virtual screening models, we identified pollutants that can modulate LPS bioactivity. This investigation revealed that bisphenol A (BPA), a chemical commonly used in numerous household items and previously implicated in obesity and cancer, effectively neutralizes LPS. In-depth mechanistic analyses showed that BPA specifically binds to the lipid A component of LPS, leading to inactivation. This interaction eliminates the immunostimulatory activity of LPS, making mice more susceptible to house dust mite (HDM)-induced allergic asthma. BPA reactivates lung epithelial cells, consequently amplifying type 2 responses to HDMs in dendritic cells. This chemical interplay provides new insights into the pathophysiology of asthma in relation to human exposure. Understanding the intricate relationships between environmental chemicals and microbial antigens, as well as their impacts on innate immunity, is critical for the development of intervention strategies to address immune disorders resulting from urbanization.

**Significance Statement**

The interplay between small molecules and microbial products, such as lipopolysaccharides (LPSs), has been relatively unexplored. In this study, we utilized extensive chemical screening to uncover that the widely employed plasticizer, bisphenol A, impedes LPS immunogenicity both in vitro and in vivo through direct binding–neutralizing actions, subsequently abrogating the protective influence of LPS on asthma development. The presence of such chemical–LPS interactions in natural environments was further corroborated by observing the neutralization effect of a chemical cocktail derived from house dust or artificially combined at human serum concentrations. This investigation unveils a mechanism and provides a predictive model for determining the impacts of environmental factors on the priming of our immune systems.

## Introduction

Asthma is a common disease worldwide, and its prevalence has been steadily increasing in developed countries and some rapidly developing nations (1). Asthma is a chronic inflammatory disease of the conducting airways that develops in response to environmental allergens such as house dust mite (HDM). It has long been considered a Th2-dominant disease of the airways. Both biological and chemical agents contribute to an individual's susceptibility to allergic asthma, and early-life exposures, in particular, can influence the risk of developing asthma later on (2). One of the factors known to play a role in asthma protection is lipopolysaccharide (LPS), a cell-wall microorganism-associated molecular pattern. Research has shown that early gestational exposure to LPS can protect against allergic sensitization through the stimulation of innate pattern recognition receptors (3). This finding is supported by epidemiological data (4, 5). Children raised in LPS-rich environments have a lower incidence of allergic asthma due to the desensitization of toll-like receptors (TLRs) (6). Furthermore, the asthma-reducing effects of LPS have also been shown in older mice (7), but this depends on a higher LPS concentration (8).

Environmental pollutants have emerged as one of the most serious threats to human health in recent decades. As environmental pollution has increased, so has the accumulation of pollutants in the human body, such as plasticizers, personal care products, and perfluoroalkyl chemicals (9). There is growing evidence suggesting that these environmental chemicals can influence the risk of immune-mediated diseases, including immunosuppression and asthma (10). Recent studies reveal that when these pollutants surpass a certain threshold in the body, they can stimulate receptors such as the estrogen receptor (ER), the aryl hydrocarbon receptor, and the peroxisome proliferator-activated receptor (11). However, it remains unclear how chronic exposure to low levels of pollutants translates into immunotoxic potential and how co-exposure to microbial LPS impacts immune responses. This lack of understanding is notable, considering the significance of mixed-toxicity exposure, particularly in infants and toddlers with potentially underdeveloped immune systems (e.g. higher asthma incidence and increase during last decades) and increased exposure to pollutants due to more frequent hand-to-mouth activity (i.e. higher exposure) or lower biotransformation capacity. To better grasp the interplay between environmental pollutants and immune system responses, further research is needed.

In our previous study, we found that one major perfluorinated compound, perfluorooctane sulfonic acid with a low dose can bind to indoor antigens (e.g. HDM der p 1), leading to reduced immune response (12). With the hypothesis that environmental chemicals can interact and modify the release or activity of microbial components, in the current study, we greatly expanded the study by screening the effects of chemicals on the release and biological activity of *Escherichia coli* LPS using Limulus lysate test screens and virtual screening models. By assessing over 1,000 compounds, the research identified bisphenol A (BPA)—a common endocrine disruptor—as a potent suppressor of LPS-mediated immune stimulation. To establish the relationship between LPS and BPA, various biophysical, molecular, and computational approaches were employed. Furthermore, both in vitro and in vivo experiments were conducted on healthy and diseased mouse models and cells to evaluate the influence of BPA on LPS-mediated immune stimulation and asthma protection. In conclusion, this study suggests that the development of human asthma may be influenced by a complex interaction of environmental factors, such as exposure to toxicants like BPA, and microorganisms. Further research into these interconnections could contribute to a better understanding of asthma pathogenesis and inform potential preventive or therapeutic strategies.

## Results

### Common environmental chemicals reduce *E. coli* LPS release via a direct neutralization effect

**Fig. 1.**
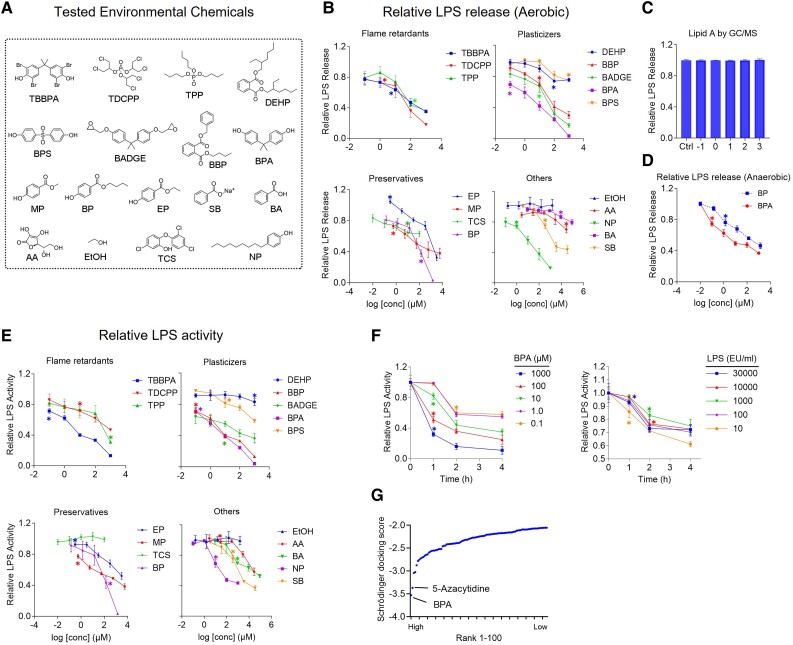
Environmental chemicals neutralize LPS activity. A) The structures of the chemicals tested. B) *E. coli* LPS release under aerobic conditions stimulated with various doses of chemicals. BBP, benzyl butyl phthalate; DEHP, bis(2-ethylhexyl) phthalate; MP, methyl paraben; EP, ethyl paraben; SB, sodium benzoate; BA, benzoic acid; TCS, triclosan; AA, ascorbic acid; NP, nonylphenol; EtOH, ethanol. C) Gas chromatography-mass spectrometry (GC/MS) analysis of lipid A levels in LPS from the secretome. D) Anaerobically stimulated *E. coli* LPS release with BPA and BP. E) *E. coli* LPS release activity stimulated with various doses of chemicals. F) Validity and dynamics of LPS neutralization by BPA. G) Docking activity predicted by Schrodinger software. The highest docking scores were aligned with BPA. Data are presented as the mean ± SEM of triplicates compared with the vehicle control (0.1% DMSO). * indicates the lowest concentration with statistically significant differences (*P* < 0.05, ANOVA).

The effect of common environmental chemicals on LPS release from *E. coli* under aerobic conditions was first investigated with the screening of 17 common immune-relevant environmental chemicals (Fig. 1A, Tables S1 and S2), such as five plasticizers, four personal care products, three flame retardants, and five others. Interestingly, 16 of 17 chemicals reduced LPS levels (Fig. 1B). BPA, a common plasticizer, was the most potent chemical, reducing LPS levels by 96.4% with a half maximal effective concentration (EC_50_) of 3.20 μM, equivalent to ∼731 ng/mL. There was no change in total secreted LPS levels in the presence of BPA (Fig. 1C), indicating that biosynthesis and secretion were unaffected. The effect of two compounds, BPA and butyl paraben (BP), on *E. coli* LPS release under anaerobic conditions was further investigated (Fig. 1D), and a similar reduction was observed.

To determine whether the chemicals tested act as LPS neutralizers, they were used to treat *E. coli* LPS, with 15 of them showing LPS neutralization effects (Fig. 1E). To understand the impact of pollutants on LPS neutralization in humans, we conducted a thorough literature search of tested chemicals in human biological fluids (Table S3). Notably, the EC_10_ value of tetrabromo BPA (TBBPA), tris(1,3-dichloro-2-propyl)phosphate (TDCPP), triphenyl phosphate (TPP), BPA diglycidyl ether (BADGE), BPA, and BP were similar to their concentration in human biological fluids. BPA remains the most potent chemical with the EC_50_ being 2.29 μM, which is approximately equivalent to 523 ng/mL. *Escherichia coli* LPS treatment with increasing BPA concentrations resulted in time- and dose-dependent LPS neutralization (Fig. 1F). Furthermore, a low BPA dose (10 μM) effectively neutralized LPS at environmentally relevant concentrations (10–30,000 EU/mL).

Despite the paucity of data on small-molecule LPS inhibitors in the literature, the binding affinity of LPS with 1,363 compounds from a Food and Drug Administration–approved drug library (Fig. 1G) was further screened using grid generation (Schrodinger module). The LPS–BPA was found to have the highest docking score value, followed by 5-azacitidine, a known inhibitor of LPS-induced acute respiratory distress syndrome (13).

### BPA diminishes the prophylactic effect of low-dose LPS in an experimental asthma model

**Fig. 2.**
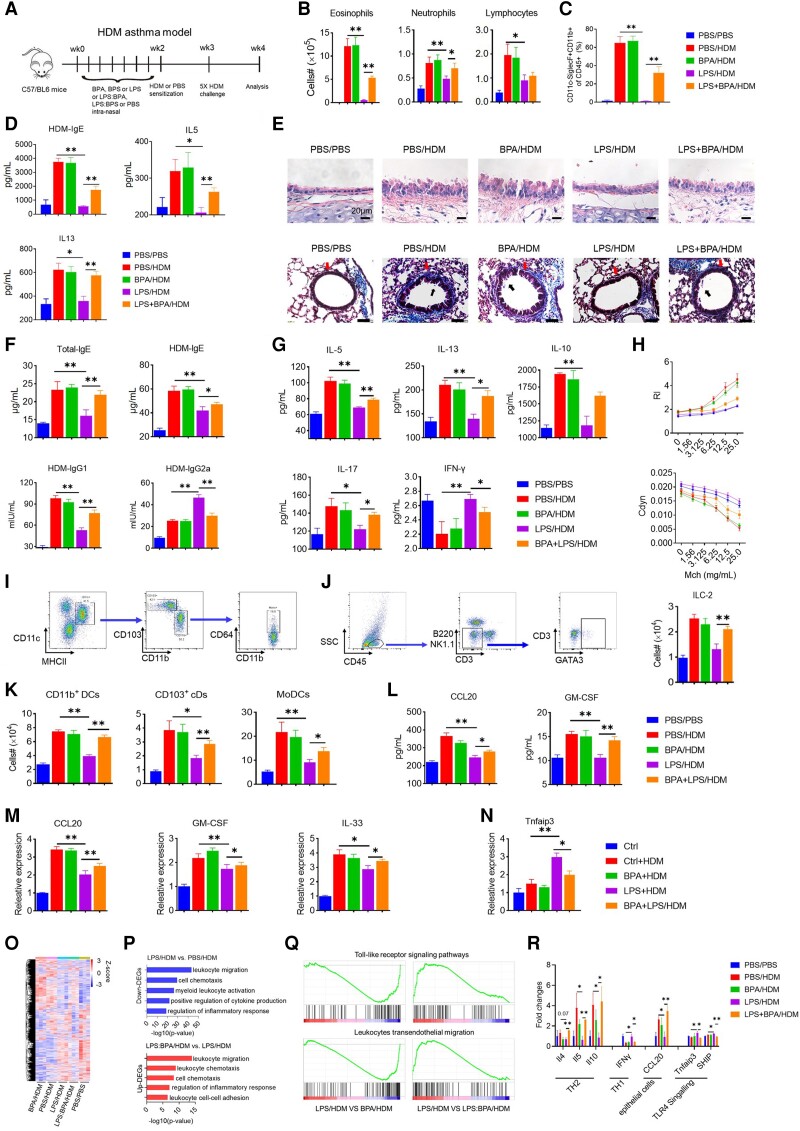
Early BPA exposure inhibits LPS-induced asthma protection. A) An experimental setup illustrates the dosing regimen of LPS, LPS/bisphenol (BPA) or BPS complex, and the various controls. B) Cell count in BALF. C) Frequency of CD11c^+^ and CD11b^+^ cells (gated from CD45^+^ cells). D) Enzyme-linked immunosorbent assay results for HDM-specific IgE, IL-5, and IL-13 levels in BALF. E) Hematoxylin and eosin stain of the trachea (upper) and Masson's trichrome stain (below) of the lungs. F) Serum total IgE, HDM-specific IgE, IgG1, and IgG2a levels. G) Cytokine production by MLN cells was restimulated with HDMs for 3 days in vitro. H) Lung resistance (RI) and dynamic lung compliance (CDYN) were evaluated after exposure to increasing methacholine (Mch) doses. I) Gating strategy for identifying DCs and DC subsets in mouse lungs. J) Recruitment of DC subsets to the lungs. K) Lung ILC-2 gating strategy and ILC-2 numbers. L) Chemokine/cytokine levels were measured in lung homogenates 2 h following HDM sensitization. M) Chemokine/cytokine mRNA levels were measured in sorted lung epithelial cells. N) TNFAIP3 mRNA levels were measured in sorted lung epithelial cells. O) Hierarchical cluster analysis of common DEGs in the four treatment groups compared with the control PBS group. P and Q) GO enrichment (P) and GSEA (Q) revealed DEGs between the selected groups. R) Treatment effect on immune-related gene expression levels. “HDM” and “Ctrl” stand for sham pretreatment with and without the HDM challenge, respectively. In vivo: Experiments were conducted twice on at least five animals per group. Data are presented as the mean ± SEM. Transcriptomic analysis: The data are presented as the mean of three mice per group compared with the control PBS group. *P < 0.05 and **P < 0.01 (ANOVA).

Since indoor house dust is an important source of BPA and LPS co-exposure, the effect of BPA on the protective effect of LPS exposure was further investigated in an experimental HDM-induced asthma mouse model (12). Based on the tolerable daily BPA intake of <50 μg/kg/day (14) stipulated by the United States Environmental Protection Agency, a low dose of 100 μg/kg BPA was chosen for mouse experiments. Mice were intranasally given 100 ng LPS ± 100 μg/kg BPA, or phosphate-buffered saline (PBS) every other day for 2 weeks prior to HDM sensitization and challenge (Fig. 2A). In mice, intranasal injections of LPS and BPA mimic the pathogenesis of childhood asthma. Mice could have a significant Th2-cell response and eosinophilic airway inflammation in response to HDM sensitization and subsequent challenge. Pretreatment with LPS reduced airway inflammation and Th2 responses in mice. In contrast, mice pretreated with BPA + LPS had more eosinophils and neutrophils in their bronchoalveolar lavage fluid (BALF) than those given LPS alone (Figs. 2B and C, S1, and S2). We also found higher levels of HDM-specific IgE, IL-5, and IL-13 in the BALF of LPS + BPA-pretreated mice (Fig. 2D). Lung histological examinations confirmed these findings, revealing that LPS + BPA-pretreated mice had a damaged airway epithelium and thicker collagen fiber stains on their bronchi than LPS-pretreated mice (Fig. 2E). We found elevated total IgE, HDM-specific IgE, and IgG1 (Th2 antibodies) levels and lower IgG2a (a Th1 antibody) levels in the sera of LPS and BPA-pretreated mice (Fig. 2F), indicating restored defective Th2 responses. To investigate HDM-specific TH1/Th2 responses, cells from mediastinal lymph nodes (MLNs) were restimulated in vitro with HDM allergen. LPS + BPA increased the production of Th2 cytokines (IL-5, IL-10, and IL-13), as well as a Th17 cytokine (IL-17), while reducing the production of the Th1 cytokine interferon-gamma by LPS-pretreated MLNs (Fig. 2G). LPS and BPA abrogated the LPS-induced decrease in methacholine-induced airway hyperresponsiveness in mice inoculated with HDM (Fig. 2H). These findings conclusively demonstrate that BPA or other active environmental chemicals that neutralize LPS may compromise the immunological protective action against asthma.

Mice with LPS-pretreated lung epithelial cells are resistant to HDM stimulation, resulting in decreased dendritic cell (DC) activation and Th2 responses (15, 16). Because pretreatment with LPS + BPA corrected faulty Th2 responses to HDM, we were curious to see whether DC activation was also restored during the sensitization phase; thus, lung DC numbers and activation status were assessed. LPS-pretreated animals attracted fewer CD11b^+^ conventional DCs (cDCs), CD103^+^ cDCs, and monocyte-derived DCs (Fig. 2I and J). In contrast, in LPS + BPA-pretreated animals, the LPS-induced defect in DC recruitment was attenuated. In addition, innate lymphoid cell (ILC)-2 is activated in the early stages of upstream DC sensitization (17), and LPS + BPA-pretreated mice had increased ILC-2 in the lung compared with LPS-pretreated mice (Fig. 2K). LPS + BPA reversed LPS-induced defective DC and ILC-2 activation after HDM exposure, consistent with restored Th2 responses.

Furthermore, the lung HDM-induced granulocyte-macrophage colony-stimulating factor (GM-CSF) and chemokine (C-C motif) ligand 20 (CCL20) protein levels were higher in the LPS + BPA-treated group than in the LPS-treated group (Fig. 2L). We also discovered that epithelial cells from LPS + BPA-pretreated mice had lower CCL20, GM-CSF, and IL-33 mRNA expression and higher tumor necrosis factor-alpha (TNF-α)-induced protein 3 (TNFAIP3) expression than MLN cells from LPS-pretreated mice (Fig. 2M and N). Thus, in response to HDM exposure, LPS + BPA induced lung epithelial cells to release CCL20, GM-CSF, and IL-33, indicating that the lungs of LPS + BPA-treated mice exhibited robust DC and ILC-2 responses upon HDM stimulation.

RNA sequencing was then used to determine BPA's immunomodulatory effect on LPS (Fig. 2O). Gene Ontology (GO) enrichment analysis revealed that differentially expressed genes (DEGs) were expressed at higher levels in the LPS/BPA-treated groups than in the LPS-treated group and were primarily involved in immunity and defensive responses (Fig. 2P). Gene set enrichment analysis (GSEA) identified DEG sets in the LPS/BPA-treated groups that were highly enriched in the Kyoto Encyclopedia of Genes and Genomes terms “toll-like receptor signaling pathways” and “leukocyte transendothelial migration” compared with the LPS-treated group (Fig. 2Q). The finding that the “regulation of immune response” was the pathway most strongly influenced by LPS + BPA pretreatment is supported by weighted correlation network analysis (Fig. S3).

The expression levels of these immune-related genes were also compared. Compared with the LPS-protected group, simultaneous BPA and LPS exposure significantly increased Th2 response genes while decreasing Th1 response genes (Fig. 2R). Furthermore, lung homogenates from LPS + BPA-pretreated mice contained more CCL20 than the LPS-treated group. TNFAIP3 mRNA levels in the lungs of LPS + BPA-exposed mice treated with HDM were reduced. Among these genes, *IL-5*, *IL-10*, *interferon-gamma*, and *TNFAIP3* were validated using real-time polymerase chain reaction (Fig. S4 and Table S4). Overall, the gene expression data suggested that BPA may reduce asthma protection against LPS.

To determine whether BPA's primary target ER-related pathways are involved in this immune response change, the expression of previously reported ER target genes (18) of the four groups (BPA vs. PBS and BPA + LPS vs. LPS) was examined. The findings revealed that low-dose BPA exposure had no effect on the ER pathway (Table S5).

### BPA effectively neutralizes and dissociates LPS

**Fig. 3.**
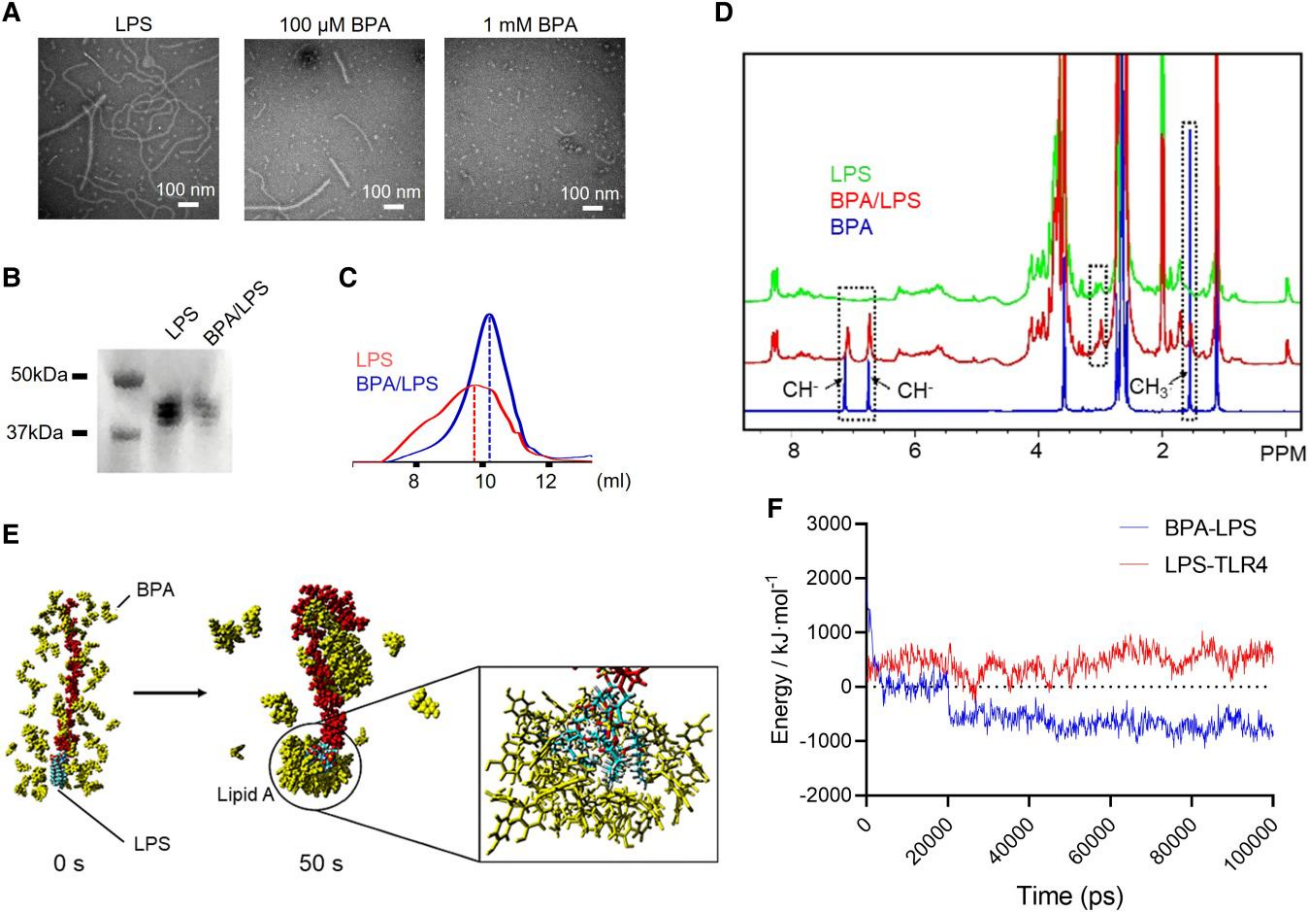
Mechanism of BPA-induced LPS neutralization. A) Transmission electron microscopy images demonstrating the effects of BPA on *E. coli* LPS. B, C, and D) Analysis of LPS and LPS–BPA mixtures using SDS–PAGE (B), GPC (C), and NMR spectroscopy (D). E) Conformational change of LPS in the presence of BPA. LPS and BPA. F) Binding energies (kJ/mol) between TLR4 and BPA.

Next, to understand how BPA neutralizes LPS, transmission electron microscopy was used to examine the effect of BPA on the structure of LPS. The results revealed that when exposed to BPA, the LPS structure was disrupted, resulting in shorter lengths (Fig. 3A). Therefore, it is plausible that BPA-mediated LPS deactivation is caused by structural integrity disruption. The LPS–BPA interaction was further investigated using sodium dodecyl-sulfate polyacrylamide gel electrophoresis (SDS–PAGE; Fig. 3B) and gel-permeation chromatography (GPC; Fig. 3C). Instead, of modulating LPS mobility, BPA reduced the LPS-induced band in SDS–PAGE. In GPC, the LPS–BPA mixture showed a peak shift of 0.28 mL corresponding to a nearly 3 kDa change. Using nuclear magnetic resonance (NMR) (Fig. 3D), sharper NMR resonances from the lipid A moiety of LPS after BPA treatment were observed, as well as noticeable differences in resonances from hydroxyphenyl and methyl groups within BPA, indicating that the lipid A moiety is a binding site for BPA. The fast protein liquid chromatography-purified LPS–BPA complex had a similar LPS neutralization effect, while the residual-free BPA in the mixture had no effect on LPS neutralization (Fig. S5), indicating that the LPS and BPA interaction was stable. Furthermore, the computational analysis revealed that BPA could bend the conformations of O-antigen polysaccharides via hydrogen bonding and make hydrophobic contacts with the lipid A moiety (Fig. 3E). Notably, the simulations revealed that the interaction between BPA and the lipid A moiety most likely interferes with TLR4 binding to LPS (Fig. 3F).

### Chemical cocktails neutralize *E. coli* LPS activity

**Fig. 4.**
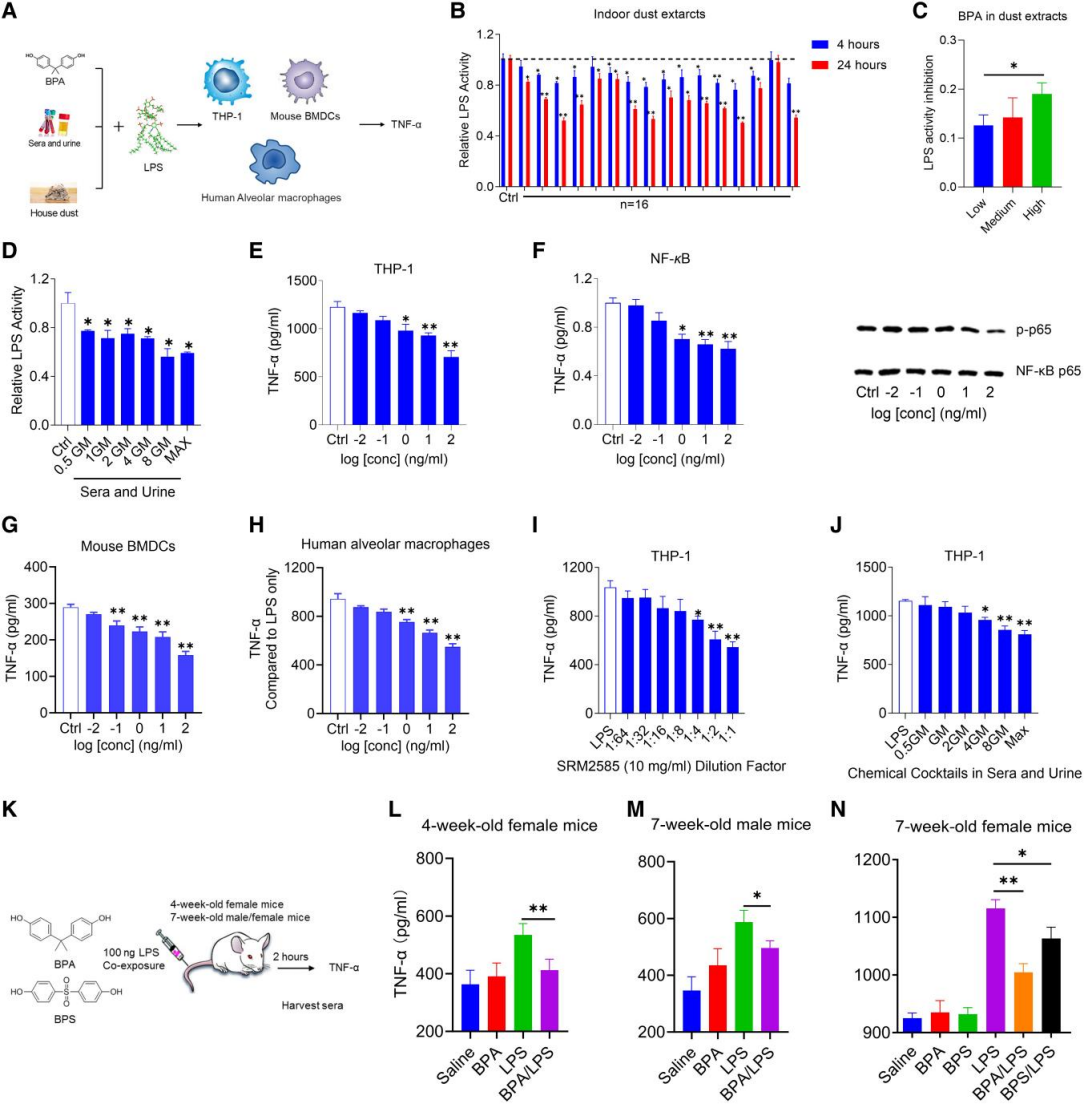
Xenobiotics suppress LPS-induced immune responses both in vitro and in vivo. A) Illustration of how chemical cocktails attenuate the immunogenicity of LPS in vitro. B) LPS neutralization by a chemical cocktail (10 mg dust/mL) derived from crude dust. C) Relationship between LPS neutralization potency and BPA levels in dust samples. D) LPS neutralization by a mixture of human-relevant xenobiotics. E) Inhibition of THP-1 cytokine production induced by LPS by BPA. F) BPA inhibited NF-κB signaling pathway activation after LPS stimulation and Western blot analysis of NF-κB p65 phosphorylation in THP-1 co-treated with LPS and BPA. G and H) The effect of BPA on cytokine production by human alveolar macrophages (G) and mouse bone marrow–derived DCs (H) treated with LPS. I and J) Inhibition of THP-1 cytokine production induced by LPS by dust extracts (I) and a human-relevant xenobiotic mixture (J). K) Experimental setup showing the dosing regimen of LPS and compounds BPA or BPS in vivo. L, M, and N) Serum cytokine levels following different treatments. In vitro: Data are presented as the mean ± SEM of triplicates compared with an *E. coli* LPS-co-treated DMSO vehicle control. “GM” and “Max” represent geometric mean and maximal level, respectively. In vivo: Data are presented as the mean ± SEM of five mice per group. *P < 0.05 and **P < 0.01 (ANOVA).

Indoor dust is a major source of indoor chemicals for humans. Therefore, it is important to investigate whether chemical cocktails derived from household dust can neutralize LPS (Fig. 4A). Chemicals were extracted from 15 dust samples and a house dust standard reference material (SRM2585) and then challenged with LPS (100 EU/mL) in vitro (Fig. 4B). After 4 h of incubation, 15 of the 16 dust extracts could moderately neutralize LPS at 10 mg dust equivalent quantity/mL. After 24 h, the highest neutralization efficiency of 50% was achieved, and SRM2585 extracts exhibited dose-dependent neutralization potency, even at concentrations as low as 0.31 mg dust equivalent quantity/mL (Fig. S6). Furthermore, the chemical extracts of SRM2585 obtained using biologically relevant digestive fluids (19, 20) demonstrated greater neutralization potency than the chemical extraction method. Some potent indoor chemicals were found in the dust. We sorted the BPA pollutant concentration from low to high and evenly divided it into three categories: low (12.7 to 23.4 μg/g, *n* = 5), medium (42.1 to 68.4 μg/g, *n* = 5), and high (83.5 to 1,657 μg/g, *n* =5). Subsequently, we analyzed the corresponding LPS neutralization data for each category. The findings revealed a suggestive positive association between the amount of BPA present in dust and LPS neutralization (Figs. 4C and S7). These findings strongly indicate that LPS neutralization by indoor chemicals has a great environmental impact.

Furthermore, to better understand the human relevance of xenobiotic exposure to LPS neutralization, an extensive literature search was conducted to identify active chemicals capable of neutralizing LPS at low-level concentrations (<100 nM) in human fluids. Chemical mixtures containing the identified xenobiotics were prepared at physiologically relevant concentrations. The LPS neutralization correlated with the concentration of chemical mixtures, with a significant effect observed at half geometric mean levels (Fig. 4D).

### BPA and chemical cocktails inhibit *E. coli* LPS immunogenicity in vitro and in vivo

To investigate the effects of indoor chemicals on LPS immunogenicity, inflammatory cytokine production by human leukemic THP-1 macrophages treated with a base dose of *E. coli* LPS and increasing BPA concentrations was measured. When combined with LPS, BPA inhibited the production of proinflammatory cytokines such as interleukin (IL)-1β and TNF-α (Figs. 4E, S8, and S9). Consistent with these findings, BPA markedly reduced LPS-mediated IL-1β mRNA upregulation, with no significant changes in the p38-MAPK, p44/42 MAPK, inflammasome-related caspase-1, and NLRP3 signaling pathways at the concentrations tested (Fig. S10). However, BPA inhibited nuclear factor kappa B (NF-κB) activity in the THP-1 cell-based luciferase assay and reduced the phospho-NF-κB p65/total NF-κB p65 ratio (Fig. 4F), confirming that BPA inhibited LPS-stimulated NF-κB activity.

To further investigate whether LPS immunogenicity was inhibited by direct neutralization, THP-1 macrophages were treated with preincubated LPS–BPA mixtures for 0, 2, or 12 h (Fig. S11). THP-1 macrophages were also pretreated with 1.0 and 100 ng/mL BPA for 2 h before being exposed to LPS (Fig. S12). Premixed LPS–BPA inhibited TNF-α production, with longer preincubation resulting in greater LPS neutralization. In contrast, pretreatment of THP-1 cells with BPA increased TNF-α production marginally more than LPS alone.

Other cell types, including primary mouse bone marrow–derived DCs, and human alveolar macrophages, were also treated to confirm BPA's neutralization effect on LPS immunogenicity, and a similar decrease in TNF-α was observed (Figs. 4G and H and S13). In addition, similar reductions in proinflammatory cytokine levels were observed in sera and urine after treatment with SRM2585 and xenobiotic cocktails (Figs. 4I and J and S14). In addition, to better understand the in vivo impact of xenobiotic exposure on LPS-stimulated responses, serum proinflammatory cytokine levels were measured after exposure to a low LPS dose in the presence of BPA and bisphenol S (BPS), a BPA replacement (Fig. 4K). An intravenous injection of LPS (100 ng) increased serum TNF-α levels. A low dose of 100 μg/kg BPA was chosen for these mouse experiments. BPA injection diminished the secretion of cytokines in vivo (Fig. 4L–N), and the anti-inflammatory effects in vivo were observed in mice of varying ages and genders. In addition, BPS also reduced LPS-induced cytokine production, although with less efficacy, which is consistent with BPS's lower in vitro neutralization potency. These results further confirmed that BPA mediated the inhibition of LPS immunogenicity through direct neutralization.

## Discussion

In this study, co-exposure of LPS to common indoor chemicals at low micromolar or nanomolar levels can actively inhibit NF-κB activation and downstream cytokines in vitro and in vivo via LPS neutralization. Many of the active compounds studied have ubiquitous environmental and human exposure. BPA, for example, is still widely used in plastic products and has been found in many environments and human biological fluids. Despite the moderate LPS neutralization effect of other active compounds, such as bis(2-ethylhexyl) phthalate, BPS, sodium benzoate, benzoic acid, and ascorbic acid, these compounds may still pose a significant risk due to their widespread use.

The current study is also the first to report that a chemical cocktail extracted from house dust or an artificial mixture that simulates xenobiotic concentrations in human biological fluids can neutralize LPS and mitigate its immunogenicity, indicating the innate existence of such an interaction. This is consistent with another recent study that found diesel exhaust particles inhibit LPS-induced asthma protection in offspring (21), substantiating our hypothesis that environmental chemicals play a direct role in mitigating immune activation triggered by microbe-derived LPS. House dust is the final sink for most indoor chemicals and is increasingly being used as a surrogate for indoor environmental quality. Infants, toddlers, and young children spend most of their time indoors and are chronically exposed to house dust via frequent hand-to-mouth behavior. The neutralization effect of dust extracts may be driven by a single active compound or by the combined effect of multiple compounds. We found that BPA levels in house dust extracts correlated with observed LPS neutralization levels. However, more research is needed to determine which components in the dust samples are the primary contributors to the LPS neutralization effect.

The identification of a mechanism by which environmental toxins act as immunomodulators is an interesting finding in this study. Using BPA as a model compound, we observed that direct binding with LPS resulted in immune neutralization, as evidenced by multiple experiments. First, there was no crosstalk between BPA and LPS in the Limulus amebocyte lysate test, which is a direct protein-binding assay. BPA alone had no effect in the absence of LPS in the test. Second, the purified complex of LPS and BPA was stable, with no effect from residual-free BPA in the mixture, implying that the LPS–BPA complex was responsible for the reduced immune activity. Third, BPA inhibition of LPS-induced TNF-α production followed a predictable time course, with longer preincubation with BPA and LPS prior to THP-1 treatment resulting in greater neutralization. Furthermore, differentiated THP-1 cells challenged with LPS after pre-exposure to BPA produced more TNF-α than LPS alone, implying that direct contact between LPS and BPA is essential for neutralization. Finally, BPA alone was found to elicit an immune response in previous in vivo and in vitro studies. BPA has been shown to increase the production of reactive oxygen species and TNF-α (22). It should be noted that previous studies used relatively high BPA concentrations that are neither environmentally nor humanly relevant. In this study, low-dose BPA exposure at relevant human serum concentrations had no effect on cytokine production. However, very low doses of BPA treatment (0.1–1.0 ng/mL, several folds lower than the GM value of human serum concentrations) significantly inhibit LPS-induced cytokine production in human immune cells. Unlike previous studies with nonphysiologically relevant BPA levels, our findings show that BPA is not immunostimulatory by itself but can regulate the immunomodulatory effects of LPS. These findings suggest that BPA and LPS have a strong direct interaction. Overall, our findings point to a novel association between environmental toxins and immunological disease (the neutralization of the key immune modulator LPS by compounds). Long-term co-exposure to pollutants and LPS may affect asthma in more complex ways than previously thought. However, crosstalk between BPA and LPS cannot be completely ruled out, because a previous study linked ER activity to NF-κB (23). When BPA levels are sufficient to activate the ER, interaction with NF-κB may be crucial. The elimination of LPS-induced asthma protection in infancy following co-exposure of mice to BPA is another significant finding of the study. Therefore, when there are confounding environmental factor interactions, early-life contact with the microbial environment cannot guarantee protection against asthma development later in life. MLNs cells from mice pretreated with a low dose of ultrapure 0111:B4 LPS produced less IL-10 when activated in vitro with HDM. These findings suggest that the decrease in HDM-induced allergy responses observed in LPS/HDM treatment was not caused by enhanced Treg-cell function (15, 24). In contrast, other studies have suggested that LPS-induced protection against allergic asthma is due to IL-10 release by monocyte-derived alveolar and interstitial macrophages (25). These discrepancies may be due to differences in the types and doses of LPS used. Notably, the LPS used in our study was derived from ultrapure *E. coli* 011:B4, which could only activate TLR4 (26). This type of LPS has been examined in mouse studies and yielded similar reduction results in IL-10 (6, 15, 27). Thus, lung interstitial macrophages failed to alter DC functions to prevent airway allergy in mice. Chronic 011:B4 LPS exposure causes lung epithelial cells to stop producing IL-33 or GM-CSF by inducing the ubiquitin-modifying enzyme TNFAIP3 (28). The latter is protected by IL-10, most likely because the LPS type is *E. coli* 055:B5, which activates both TLR4 and TLR2. TLR2 stimulation resulted in rapid IL-10 release (29). Several recent studies have demonstrated that IL-10 has a protective effect on immune regulation in response to pathogens and commensal bacteria (1). A recent study found that the protective effect of LPS is dependent on its bacterial source (3). Because the chemical structures of these two LPS molecules differ significantly, O111:B4 LPS in this study has stronger bioactivity (Fig. S15) and is more effective in inducing tolerance than 055:B5 LPS (30). This distinction could explain why low and high levels of inhaled LPS produce different Th2 and Th1 responses to allergens, respectively, in a mouse model of allergy sensitization (8, 31, 32). These findings imply that LPS airway sensitization causes asthma, which is modulated by the LPS levels and types present at the time of aeroallergen exposure. However, the precise mechanisms by which LPS causes polarized Th1 versus Th2 responses to allergens are not fully understood (33). Therefore, to better understand how BPA inhibits LPS's protective effect, 011:b4 LPS was used to develop a well-established mouse asthma model that simplifies the mechanism of mouse airway allergy.

In addition, the severity of allergic inflammation is influenced by the HDM sensitization protocol (34). In the continuous model we used, mice developed a significant Th2-cell response and eosinophilic airway inflammation following HDM sensitization and subsequent challenge. In contrast, mice exposed intermittently to HDM produced considerably more neutrophils than those exposed continuously (34). This could explain why an abundance of neutrophils can stimulate HDM uptake by lung CD11b^+^ Ly-6C^+^ DCs, increasing the risk of allergic asthma (8). Different HDM sensitization protocols may induce a different inflammatory response; however, this effect on neutrophils is limited in the continuous model because neutrophils comprise a small proportion of total cells. These discrepancies may be attributed to variations in the type and timing of antigen sensitization protocols. The antigen sensitization protocol should be carefully studied when designing experiments to investigate the underlying processes of allergic inflammation in mouse models of asthma.

One recent clinical study, which found that BPA exposure is significantly associated with increased pediatric asthma morbidity in a predominantly low-income American urban child cohort (adjusted odds ratio: 1.40, 95% CI: 1.02–1.92) (35). However, there is no exact mechanistic explanation for this association; such BPA–LPS interaction may provide a new potential explanation supporting the role of BPA postnatal exposure in asthma and wheezing pathological processes. Increased consumption of plasticized products, food packaging, and personal care products associated with urbanization will result in unprecedented chemical exposure, potentially reducing LPS-induced immune protection via a direct neutralizing effect. According to the “exposome” pyramid, human health is influenced by environmental exposure to both biotics (e.g. microbes) and abiotics (e.g. chemicals) (36). A recent study has identified 1,561 chemicals in human blood (37), but little is known about how they affect human health. Although the direct health effects of environmental chemical cocktails and microorganisms have been studied separately, their potential interactions have not been investigated. This is one of the first studies to examine the complex interaction of these exposomic components. Our findings revealed that allergic sensitization and asthma are caused by pathogens or microorganisms, as well as a complex interaction with various environmental factors (e.g. chemicals vs. LPS). To expand on our findings, it would be beneficial to explore the connections association between chemical–LPS interactions and human health and human exposure during other critical window periods such as aging processes, as well as whether environmental factors interact similarly with other asthma allergens such as HDMs, pets (animal dander), pollen, and mold (38). Understanding the interaction between environmental chemicals and microbial components, as well as their impact on asthma, will be crucial in developing primary prevention strategies against allergy and asthma risks associated with urbanization.

## Methods

### Bacterial culture and pure LPS treatment with xenobiotics

*Escherichia coli* K12-MG1655 was used as the model gram-negative bacterium for this study. Prior to experiments, bacteria were inoculated in a pyrogen-free glass tube containing LB and incubated for 12 h at 37°C under aerobic conditions. For anaerobic experiments, *E. coli* were grown in Reinforced Clostridial Medium for 12 h at 37°C. Following preincubation, bacterial suspensions were aliquoted into 15 mL glass tubes and exposed to the indicated doses of chemicals for 24 h at 25°C. We have run the bacterial growth monitoring prior to the concentration design, and all these doses are below the antibacterial concentration of the chemical for *E. coli*. LPS samples were measured using a LAL assay. To investigate whether chemical-induced reduction in LPS was caused by neutralization, LPS (100 ng/mL) from *E. coli* 055:B5 was incubated with tested chemicals for 4 h at 25°C as detailed above. BPA was used as a model compound to understand the dynamics, kinetics, and mechanism of LPS neutralization, and a chemical cocktail, either isolated from house dust or artificially mixed at human serum level, was also investigated their LPS neutralizing effect, as well as a virtual screening model was used to predict screen large libraries of chemicals binding to LPS. The detailed procedures are described in Supplementary material.

### Immune stimulation in vitro assays

THP-1-derived macrophages were exposed to test chemicals, dust samples, and a human-relevant chemical cocktail or dimethyl sulfoxide (DMSO; 0.1% final concentration) as the vehicle control for 24 h. Concentrations of IL-1β, and TNF-α in culture supernatants were determined using ELISA kits. THP-1-Lucia NF-κB cells were used to investigate the effect of BPA on LPS-induced NF-κB activation. Quantitative RT-PCR and Western blot were performed to measure IL-1β, CASP1, and NLRP3, and the activation states of p65, p38-MAPK, and ERK, respectively, in THP-1 macrophages. In addition, mouse BMDCs and human alveolar macrophages were also treated with BPA, LPS, LPS + BPA, and vehicle controls. The detailed procedures are described in Supplementary material.

### Mouse asthma model induced by HDM

All animal studies were approved by the Committee on the Use of Live Animals for Teaching and Research, Shenzhen Institutes of Advanced Technology, Chinese Academy of Sciences (SIAT-IACUC-181225-YYS-LL-A0550). All experiments were conducted following the approved guidelines and regulations. Female (5-week-old early-weaned) C57BL/6 mice were housed in BPA-free cages and maintained on a 12-h light-dark cycle. The mice were given ad libitum feed with minimal phytoestrogens and drinking water in BPA-free PET bottles. The mouse asthma model was modified from one previous study (12), wherein 1 μg of HDM was administered intratracheally in the anesthetized mice on day 0. Prior to the challenge, mice were pretreated every other day with 100 μg/kg of BPA, 100 ng of LPS, or LPS in combination with BPA delivered intranasally from 14 days prior to the first HDM challenge. In addition, Bronchoalveolar lavage analysis, airway hyperresponsiveness, flow cytometric analysis, cytokine assays, lung histology, and transcriptomic analyses are described in Supplementary material.

### Statistical analysis

For in vitro assays, all data are representative of three or more independent experiments and are presented as the mean (and SEM) of triplicate assessments. For in vivo animal studies, two batches of experiments were conducted. Differences between groups were examined for statistical significance using Student'’s *t* test or a one-factor ANOVA with Duncan’s post-hoc test. If the data were not normally distributed or if there was a violation of the assumption of homogeneity of variance, alternative tests, such as nonparametric tests, may have been used instead. The significance level was set at *P < 0.05 and **P < 0.01. EC_10_/_50_ values were estimated from LPS neutralization data curves using a 3-parameter sigmoidal dose–response model in GraphPad Prism 8.0.

## Supplementary Material

pgad312_Supplementary_Data

## Data Availability

All study data are included in the article and Supplementary material.
