# Supplementary material for: A missing jigsaw within the hygiene hypothesis: Low-dose bisphenol A exposure attenuates lipopolysaccharide-induced asthma protection
Source: PNAS Nexus. 2023 Nov 7;2(11):pgad312. doi: 10.1093/pnasnexus/pgad312 (PMC10635653; doi:10.1093/pnasnexus/pgad312)
Supplement: pgad312_Supplementary_Data [file pgad312_supplementary_data.docx]

Supplementary Information for

**A Missing Jigsaw within the Hygiene Hypothesis: Low-Dose Bisphenol A Exposure Attenuates LPS-Induced Asthma Protection**

Mengjing Wang^1,2#^, Jing Qu^3#^, Junjie Yang^2^, Tian Zhang^4^, Wei Ren Tan^4^, Shumin Liao^5^, Xing Chen^6^, Yingzi Liu^7^, Xiang Long^8^, Xue Li^6^, Yun Xia^4^, Nguan Soon Tan^4^, Liang Li^5^*, Mingliang Fang^1^*

**Affiliations:**

^1^Department of Environmental Science and Engineering, Fudan University, Shanghai 200433, China

^2^School of Civil and Environmental Engineering, Nanyang Technological University, 639798 Singapore, Singapore.

^3^Department of Pathogen Biology, Shenzhen Center for Disease Control and Prevention, Shenzhen, China.

^4^Lee Kong Chian School of Medicine, Nanyang Technological University, 639798 Singapore, Singapore.

^5^School of Medicine, Southern University of Science and Technology, Shenzhen, China.

^6^Institute of Mass Spectrometer and Atmospheric Environment, Jinan University, 510632 Guangzhou, China.

^7^Intervention and Cell Therapy Center, Peking University Shenzhen Hospital, Shenzhen, Guangdong, China.

^8^Department of Respiratory Medicine and Critical Care, Peking University Shenzhen Hospital.

^#^These authors contributed equally to this work.

*Correspondence and requests for materials should be addressed to LL (email: lil@sustech.edu.cn) or to MF (email: [mlfang@ntu.edu.sg](mailto:mlfang@ntu.edu.sg)).

**This PDF file includes:**

Materials and Methods

Figures S1 to S15

Tables S1 to S5

References

Supplementary Materials and Methods

**Reagents.** The chemicals used in this study are listed in Tables S1-S2. A total of 17 chemicals were selected, comprising of 5 plasticizers, 4 personal care products, 3 flame retardants, and 5 other compounds (1-3). All the tested chemicals were procured from commercial suppliers with a purity of >97%. Stock solutions, except for sodium benzoate, were prepared in 100% DMSO and stored at -40°C until use. Sodium benzoate was dissolved in LPS-free water. Lipopolysaccharide (LPS) from *E. coli* 055:B5 and *E. coli* O111:B4 were procured from Sigma-Aldrich (Singapore). Ultrapure LPS was obtained from Beyotime (Shanghai, China), while house dust mite (HDM) extracts were procured from Greer Laboratories (Lenoir, NC). All solvents used, which were HPLC-grade, were obtained from Fisher Chemicals (Fair Lawn, NJ).

**Pure LPS treatment with chemicals.** In order to investigate the kinetics and dynamics of LPS neutralization, we utilized bisphenol A (BPA) as a representative compound to titrate pure *E. coli* LPS O55:B5 (30,000 EU/ml). Bacteria were incubated with BPA at concentrations ranging from 0.1 to 1,000 μM for a period of 4 hours at 25 °C. Furthermore, low-dose BPA (10 μM) was employed in combination with pure *E. coli* LPS O55:B5 concentrations ranging from 10-30,000 EU/ml. Bioactivity was measured at 1, 2, and 4 hours.

To investigate the environmental relevance of chemical interactions with LPS, pure LPS derived from *E. coli* was subjected to treatment with environmentally relevant house dust extracts. The extracts were diluted in pyrogen-free water to a final concentration of 10 mg dust equivalent quantity/ml. Samples for LPS analysis were collected immediately after treatment, and after 4 and 24 hours of incubation at 25 °C. The neutralization dose-response was quantified using the standard reference material SRM2585. To evaluate the effect of a human-relevant environmental chemical cocktail on LPS activity, an extensive literature search was conducted to identify chemicals that induce LPS neutralization at very low concentrations (<100 nM) in human serum or urine. The geometric mean (GM) and maximal concentrations of the identified chemicals were summarized, and a chemical mixture was prepared to mimic their concentrations in human biological fluid. Pure *E. coli* LPS (30,000 EU/ml) was then treated with varying concentrations of the chemical cocktail for 24 hours at 25 °C.

**Dust sample collection and processing.** We conducted LPS neutralization assays using extracts from indoor dust samples (n=15) and house dust Standard Reference Material (SRM) 2585 obtained from the National Institute of Standards and Technology (Gaithersburg, MD). The dust samples were collected from 15 common households in Durham, NC, US in 2017 and were generously provided by Dr. Heather Stapleton's laboratory at Duke University. The collection method has been described in detail in our previous studies (4-6). For the chemical extraction method, we sequentially extracted approximately 50 mg of dust with acetone:hexane (50:50, v/v), methanol, and water via sonication. As shown in Figure S2, this multistep extraction method, which is a modification of previously published methods (7, 8), was employed to recover a broad range of chemical classes with biological relevance. The resulting extracts were pooled, concentrated, and reconstituted in 100 μl DMSO for use. We prepared three procedural blanks with pure solvents and processed them in parallel. None of the procedural blank samples exhibited any LPS neutralization.

**Analysis of potent chemical levels in dust samples.** To investigate the active compounds present in the dust extracts, we measured the levels of several potent LPS neutralizers, including BPA, BPS, and BADGE and its derivatives (BADGE-H_2_O and BADGE-2H_2_O, BADGE-H_2_OHCl, BADGE-HCl, and BADGE-2HCl). The dust extract was spiked with ^13^C_12_-BPA and d6-BADGE and reconstituted in methanol prior to instrumental analysis. Separation and detection of the analytes were carried out using an Agilent 1290 Infinity II Series HPLC interfaced with an Agilent 6460 electrospray triple quadrupole mass spectrometer (ESI-MS/MS) in multiple reaction monitoring modes (MRM). Five μl of the sample was injected into an analytical column (Atlantis T3, 3 µm 2.1 ×100 mm, column, Waters Corporation, Milford, USA) coupled with Atlantis T3 Vanguard (3 µm, 2.1×5 mm). The mobile phase consisted of 10% methanol in Milli-Q water with 2 mM ammonium acetate (A) and 100% methanol (B). A gradient elution was used for separation at a flow rate of 0.4 mL/min, where 50% B was used for the first 5 minutes, followed by an increase to 70% B at 20 minutes. The column was then equilibrated for 3 minutes using 50% B.

**LPS quantitation.** To ensure accurate quantitation, the samples were handled with care and processed using rigorous procedures. Samples for quantification were immediately placed on ice and diluted in pyrogen-free water containing 0.05% Tween 20. Following centrifugation at 500 ×g for 10 min, the resulting supernatant was frozen at -20 °C until analysis. Quantification of LPS was carried out using the limulus amebocyte lysate (LAL) test, which measures the activity of LPS in the sample. Specifically, the LPS-activated protease cleaves the substrate, producing the chromogenic product p-nitroaniline.

For LPS levels in indoor dust, approximately 50 mg of dust was extracted using a rigorous procedure. The dust was vortexed for 1 hour in 10 mL of pyrogen-free water containing 0.05% Tween 20. The solutions were then centrifuged at 500 ×g for 10 minutes, and the supernatants were used for LPS quantification. Control samples were also included, in which blank glass tubes were extracted and analyzed. To ensure precision in the results, the values obtained from the control samples were subtracted from the sample values. The LPS levels in SRM2585 were approximately 6 EU/mg.

**LPS quantification using gas chromatography and mass spectrometry (GC/MS).** The absolute quantity of LPS in the secretome was determined through direct measurement of 3-hydroxy fatty acids from lipid A by GC/MS, which was modified from previously published methods (9, 10). In brief, each filtrate sample in growth medium (500 μl) was prepared in triplicate and then hydrolyzed with 500 μl of 10 M NaOH for 30 minutes. The samples were subsequently acidified with 2 mL of 6 M HCl. Next, the samples were extracted twice with 3 mL of ethyl acetate, dried down under nitrogen at 37 °C, and derivatized with 100 μl of N,O-bis(trimethylsilyl) triﬂuoroa-cetamide with 1% trimethylchlorosilane for an hour at 80 °C. The derivatized samples were analyzed using an Agilent 5890 series II system with an HP-5MS capillary column coupled with a 5973 MS detector. The analytical run conditions started with an initial oven temperature of 80 °C for 5 min, then was programmed to increase 3.8 °C/min to 200 °C, and finally to 290 °C with a ramp of 15°C/min and remained for 6 min. Quantitation was performed in single ion monitoring (SIM) using the characteristic ion of m/z 233 for the 3-hydroxy fatty acid fragment. Laboratory and procedural blanks were both prepared and subtracted for the final quantification.

**Docking-Based Virtual Screening.** In this study, we employed the FDA-approved drug library, consisting of 1,363 compounds, for virtual screening. The Protein Preparation Wizard module of Schrodinger software was utilized to pre-process the protein crystal structure obtained from the Protein Data Bank (11) (PDB ID 6S8H) (11, 12) and prepare it for docking analysis. The LigPrep module was then employed to convert 2D structures of the compounds into 3D structures under the OPLS force field, with salt molecules removed (13). The grid generation module was employed to create a grid file, which determined the pocket's center where the six saturated hydrocarbon chains met during docking and the length of the restricted compound was less than 20 Å. Schrodinger software provides three precision algorithms (HTVS, SP, and XP) that were used in the docking process. Based on model testing, all three algorithms were utilized in this study. Docking accuracy improved over time, allowing for the selection of the top 20% of compounds. Gradient screening was then employed in the subsequent round of docking to identify the final top 100 compounds.

**LPS structure characterization using a transmission electron microscope (TEM).** For the purpose of investigating the impact of BPA on the structure of LPS, a concentration of 5.0 mg/mL of *E. coli* O55:B5 LPS in 0.1 M Tris-HCl (pH 7.2) was subjected to 1 mM and 100 μm BPA. Following incubation at 25°C for a duration of 4 hours, a sample volume of 4 μL was placed on a carbon-coated copper TEM grid that was glow-discharged, and was subsequently stained using a solution of 2% (v/v) uranyl acetate. Electron micrographs were captured using a Tecnai spirit T12 transmission electron microscope (FEI, Eindhoven, Netherlands) which was equipped with a 4 K Eagle CCD camera and was operated at a voltage of 120 kV.

**Proton Nuclear Magnetic Resonance (^1^H-NMR) structure determination.** To investigate the interaction of BPA with LPS, a solution containing 5.0 mg/ml *E. coli* O55:B5 LPS in 0.1 M Tris-HCl (pH 7.2) was exposed to 1 mM BPA for 4 hours at 25 °C. The interaction between LPS and BPA was analyzed by measuring the chemical shifts between their resonances. To this end, deuterium oxide (D2O) was added to the mixture at a final concentration of 5%. The data were collected using a Bruker Avance 800 MHz spectrometer equipped with a TXI cryogenic probe at 25 °C. The 1H resonance assignments were obtained using 3D HNCACB (14). All spectra were processed using NMRPipe/NMRDraw (15) and the data were analyzed using Sparky.

**LPS silver staining.** LPS from *E. coli* O55:B5 was prepared in LPS-free water to a concentration of 2.0 μg/ml and incubated with 1 mM of BPA for 4 hours at 25°C. The resulting LPS:BPA mixture, BPA alone, and pure LPS were loaded onto an SDS-PAGE gel and electrophoresed in 1× MOPS running buffer. LPS bands were detected by silver staining as follows: (i) the gel was placed in fixation solution, which was prepared by mixing 200 mL of water, 50 mL of isopropyl alcohol, and 14 mL of acetic acid, and kept at 4°C; (ii) the gel was transferred to oxidation solution, which was prepared by mixing 150 mL of water, 1.05 g of periodic acid, 4 mL of ethanol, and 500 μl of acetic acid, and agitated gently for 5 minutes; (iii) the gel was washed in 200 μl of ddH_2_O for 30 minutes and repeated 3 times; (iv) the gel was stained with fresh staining reagent, which was prepared by mixing 50 mL of water, 2 mL of 1 M NaOH, 2.6 mL of ammonium hydroxide, and 5 mL of water containing 1 g of AgNO_3_; (v) the gel was washed in ddH_2_O for 10 minutes and repeated 4 times; (vi) developing solution, which was prepared by mixing 200 mL of water, 2 mL of 1% citric acid, and 100 μl of formaldehyde, was added to the gel and allowed to develop for 10-20 minutes until the LPS bands appeared slowly and the background of the gel was yellow; (vii) the development was stopped by washing the gel in stop solution, which was prepared by mixing 200 mL of water and 1.6 mL of acetic acid; and (viii) the gel was rinsed in ddH_2_O and stored in the dark at 4°C.

**Molecular weight estimation using gel permeation chromatography.** To analyze the BPA-LPS mixture, BPA alone, and pure LPS, gel permeation chromatography (GPC) was conducted at room temperature. A PL aquagel-OH 20 column (5 μm, 300 × 7.5 mm) was used, connected to a PL aquagel-OH guard column (5 µm, 50 × 7.5 mm). The samples were eluted with HPLC water as the mobile phase, at a flow rate of 0.7 ml min^-1^.

**Molecular dynamics simulation**. Simulations were performed using GROMACS 5.1.4 software with the CHARMM36 force field and the flexible simple point-charge water model. The initial structures were placed in a periodic water box and neutralized with counter ions. Simulations were conducted in the NPT ensemble, while long-range electrostatics were computed using Particlemesh Ewald summation. Temperature was weakly coupled to T=298K at a coupling time of 0.1 ps. The LINCS algorithm was employed to constrain bond lengths, while pressure was weakly coupled to 1 bar at a coupling time of 0.5 ps and compressibility of 4.5e-5 bar. A force constant of 5000 was set for distance restraints during equilibration. Two systems underwent 50 ns simulations, and all images were generated using YASARA view 16.7.22.

***In vitro* gut reactor system set-up.** A compact chemostat system, capable of controlling temperature, dissolved oxygen (DO), flow rate and pH, was established with a maintained working volume of 2 L and a residence time of 2.8 days. The temperature control unit, attached to a surrounding heating stripe, was utilized in order to manage the temperature at 37°C. Subsequent to autoclaving the entire reactor chamber and medium for sterilization, the medium was flushed with N2 via the N2 purge inlet, that included a 0.22 μm gas filter, for a period of not less than an hour. An excellent fecal sample was then transferred to an anaerobic chamber within 1 hour, and roughly 20 g of the sample was mixed with the growth media, and then let to settle for 5 minutes for the removal of large particles. The resulted supernatant was inoculated into the pre-reduced reactor medium. The N2 purging continued for a few hours post inoculation, followed by the connection of the gas collection outlet with an empty gas bag for the collection of the gas generated. To prevent the introduction of oxygen, a gas bag filled with N2 gas was connected to the medium bottle soon after autoclaving while the medium remained hot. Furthermore, future sampling and monitoring were routinely carried out three times a week to measure OD600, chemical oxygen demand (COD), and short-chain fatty acid (SCFA).

To determine the potential neutralization capacity of bisphenol A (BPA) against the total lipopolysaccharides (LPS) in bacterial cultures, filtrates were obtained by passing the cultures through a 0.2 μm Acrodisc syringe filter (Gelman Laboratory, MI). The filtrates were then subjected to incubation with the tested chemicals for a period of 4 hours at 25°C, as previously specified. Total LPS was employed as a positive control in the analysis.

**THP-1 culture and treatments.** THP-1 macrophages, obtained from ATCC TIB-202, were cultured in a medium of RPMI1640 supplemented with 10% fetal bovine serum and 2mM L-glutamine in a controlled environment of 5% CO2 at 37 °C. THP-1 monocytes were differentiated into macrophages by stimulation with 100 ng/ml phorbol 12-myristate 13-acetate (PMA) for a duration of 48 hours. Post-differentiation, THP-1 macrophages were cultured in standard medium. Subsequently, THP-1-derived macrophages were exposed to various test chemicals, dust samples, and a human relevant chemical cocktail with the addition of: i) no other compounds, ii) 100 ng/ml E. coli 055:B5 LPS, iii) 100 ng/ml BPA or iv) a mixture of LPS and BPA for a duration of 24 h. The LPS:BPA mixture was prepared in PBS and incubated for 0, 2 and 12 hours at 4 °C. The concentrations of interleukin (IL)-1β, and tumor necrosis factor (TNF)-alpha in the culture supernatants were assessed using ELISA kits procured from Wuhan Biotech Co, Ltd, China, following the manufacturer's instructions.

To evaluate the impact of BPA exposure on the activation potential of THP-1 macrophages, the cells were treated with LPS after BPA exposure. Following exposure to 1.0 ng/ml or 100 ng/ml BPA for a duration of 6 hours, the macrophages were thoroughly washed. The cells were then exposed to 100 ng/ml LPS-supplemented culture medium for the next 2 hours, and their culture supernatants were collected for subsequent cytokine production analysis. A blank control, vehicle control, LPS control, and BPA control groups were run simultaneously. The levels of TNF-α in culture supernatants were measured using ELISA kits.

**Human Alveolar Macrophages (HAM).** Human alveolar macrophages (HAM) procured from iCell Bioscience (iCell Bioscience Inc, Shanghai, China) were cultured in RPMI 1640 medium supplemented with 10% heat-inactivated fetal calf serum, 2 mM L-glutamine, 100 U/ml penicillin, and 100 µg/ml streptomycin. The cultures were maintained at 37°C in a 5% CO2 and 95% air environment. The cells were stimulated for 24 h with 10 ng/ml of LPS, either alone or in combination with a bisphenol A (BPA). A culture medium or vehicle control group (0.1% DMSO in culture medium) was prepared as appropriate, and LPS served as a positive control. Using ELISA kits, the concentrations of TNF-α in the culture supernatants were quantified.

**Mouse BMDC Isolation and treatments.** Female C57BL/6J mice of wild type, aged 8-9 weeks (NTU, ARF), were housed in a light-dark cycle of 12:12 and a temperature-controlled environment of 22±1 °C. They were provided with standard mouse chow diet and ad libitum water supply. Following euthanasia, bone marrow-derived dendritic cells (BMDCs) were isolated according to Lutz et al. (16). In brief, bone marrow cells were collected from femurs and tibias and cultured in RPMI with 10% fetal bovine serum (FBS) and 50 μM 2-mercaptoethanol. For the first 6 days, 20 ng/ml GM-CSF (Peprotech, U.S.) was added to the medium with a medium change on day 3 at the same concentration. On day 6 and 8, immature DCs were co-stimulated with 10 ng/ml GM-CSF and 10 ng/ml IL-4 (Miltenyi Biotech, Germany) for maturation. On day 10, non-adhered mature DCs were collected and seeded at 5 x 10^3^ cells per well and cultured in a 96-well culture plate with 10% FBS RPMI and 50 μM 2-mercaptoethanol. Following treatment with BPA and LPS for respective time periods, supernatants were collected and subjected to ELISA assays.

**Detection of mature BMDCs cell surface markers via Flow Cytometry.** The identification of mature bone marrow-derived dendritic cells (BMDCs) was confirmed using flow cytometry on Day 10. Specifically, BMDCs were harvested, quantified, and subjected to staining with allophycocyanin-conjugated anti-CD11b+ and phycoerythrin-conjugated anti-MHCII+ cell surface markers at a dilution of 1:100 from Miltenyi Biotech in Germany, followed by a 30-minute incubation at 4 °C. The cell samples were then washed with PBS and scrutinized with the BD Accuri™ C6 Plus (BD Biosciences, U.S.) for determination of DC percentage. Further characterization was performed using FlowJo v10.0.7 (FlowJo LLC, U.S.).

**MTT assay.** After exposure to either LPS, BPA, or a combination of LPS and BPA for a period of either 24 or 48 hours, MTT was introduced into the culture medium at a concentration of 0.5 mg/ml. Subsequently, microplates were incubated at 37°C for 4 hours, followed by a centrifugation step at 500 ×g for 20 minutes. The supernatant was eliminated, and the intracellular purple-colored formazan crystals were dissolved using DMSO. Finally, the absorbance was recorded at a wavelength of 570 nm OD.

**NF-*κ*B reporter assay.** THP1-Lucia™ NF-κB cells from Invivogen (US) were utilized to explore the effects of bisphenol A (BPA) and LPS on NF-κB activation in differentiated monocytes. Cells were seeded at a density of 4.2 × 10^5^ cells/well in 6-well plates and allowed to differentiate for 72 hours. Following a 24-hour incubation in PMA-free media to induce monocyte differentiation, cells were preincubated with varying concentrations of BPA (0.01-100 ng/ml), and 10 ng/ml LPS for a duration of 2 hours. After treatment, the cell supernatant was collected, and the Quanti-Luc™ coelenterazine-based luminescence assay reagent was used to perform a reporter gene assay following the manufacturer’s protocol. Luciferase activity was measured in a microplate reader to determine the extent of NF-κB activation.

**Quantitative RT-PCR.** Total RNA from THP-1 stimulated macrophages was extracted using Trizol (Invitrogen). Reverse transcription was performed using 1 μg of total RNA as template and Rever Tra Ace qPCR RT master mix with gDNA remover (TOYOBO). qPCR was performed using the Thunderbird SYBR qPCR mix without ROX kit (TOYOBO) on a LightCycler 96 Real-Time PCR Detection System (Roche). The PCR conditions were: 95 °C for 10 min followed by 40 cycles of 95 °C (2 sec), 60 °C (20 sec) and 70 °C (10 sec). The following Sangon Biotech (Shanghai, China) synthesized primers were used: forward 5’-TGATGGCTTATTACAGTGGCAATG-3’ and reverse 5’-GTAGTGGTGGTCGGAGATTCG-3’ for IL-1β; forward 5’-AGCTGAGGTTGACATCACAGG-3’ and reverse 5’-GTCAGAGGTCTTGTGCTCTGG-3’ for CASP1; forward 5’-GAGGAAAAGGAAGGCCGACA-3’ and reverse 5’-CCCGGCAAAAACTGGAAGTG-3’ for NLRP3.

**Western Blots.** To examine the activation states of p65, p38-MAPK and ERK with serum starvation, cells were suspended in Hank’s balanced salt solution without glucose for 3h at 5% CO_2_, 37 °C. Cells were then placed in lysis buffer containing protease inhibitor cocktail (Millipore) and phosphatase inhibitor cocktail (Sigma-Aldrich) and incubated for 30 min on ice. Total protein concentration was determined using the bicinchoninic acid protein assay kit (Pierce, Thermo Scientific, Rockford, IL). Proteins were resolved by SDS-PAGE under reducing conditions. The following primary antibodies from Cell Signaling Technology (Danvers, MA) were used: Rabbit anti phospho-NF-κB p65 (Ser536) (93H1); Rabbit anti NF-κB p65 (D14E12; Rabbit anti phospho-p44/42 MAPK (Erk1/2) (Thr202/Tyr204) (D13.14.4E); Rabbit anti p44/42 MAPK (Erk1/2) (137F5); Rabbit anti phospho-p38 MAPK (Thr180/Tyr182) (D3F9); Rabbit anti p38 MAPK (D13E1); Rabbit anti caspase-1 Antibody and Rabbit anti NLRP3 (D4D8T).

***In vivo* LPS challenge and serum cytokine detection.** All animal experimentation was performed with the approval of the Institutional Animal Care and Use Committee (IACUC) at Nanyang Technological University, Singapore and the Animal Ethics Committee of the Research Center for Eco-Environmental Sciences, Chinese Academy of Sciences (AEWC-RCEES-2021001) in accordance with established protocols. To assess the relevance of these findings to humans, cohorts of male and female C57BL/6J mice were assigned to treatment groups and injected intravenously with either a low dose (100 ng) of *E. coli* O55:B5 LPS with or without BPA (100 μg/kg) at 4 and 7 weeks of age, respectively. Female mice were chosen for the BPS treatment group, as they demonstrate a stronger immune response compared to males, and received a dose of 100 μg/kg. Prior to administration, LPS was carefully prepared in sterile saline. After a 2-hour incubation period, mice were euthanized and blood samples collected by cardiac puncture. Serum was obtained by centrifugation of heparin-treated blood, and aliquots of samples were stored at -80°C for subsequent analysis of murine TNF-α. Administration of sterile saline served as a negative control in these experiments.

**Mouse asthma model induced by house dust mite (HDM).** Female C57BL/6 mice, which were early-weaned at 5 weeks of age, were procured and housed in cages that did not contain bisphenol A (BPA). The mice were subjected to a 12-hour light-dark cycle and were provided with ad libitum feed that contained minimal levels of phytoestrogens. Furthermore, the mice were provided with drinking water that was stored in Polyethylene terephthalate (PET) bottles that did not contain BPA. The asthma model in mice was adapted from previously conducted research works (21, 22), wherein 1 μg of House Dust Mite (HDM) was administered intratracheally in the anesthetized mice on day 0. European Food Safety Association (EFSA) decreased this TDI dose from 50 μg/kg BW/day to 4 μg/kg BW/day in 2015 (17). However, according to the United States Environmental Protection Agency (EPA), BPA's reference dose is 50 μg/kg BW/day. According to the toxicological experiments conducted on BPA, the definition of low-dose BPA ranges from 2.5-2700 μg/kg BW/day (18). We have finally chosen to study a slightly higher concentration that is double the EPA's standard (100 μg/kg BW/day) with additional safety margin. Thus prior to the challenge, mice were pre-treated every other day with 100 μg/kg of BPA, 100ng of Lipopolysaccharides (LPS), or LPS in combination with BPA delivered intranasally from 14 days prior to the first HDM challenge. The experimental control group of mice received only PBS by intranasal administration. The mice were given daily 10-μg doses of HDM for 5 consecutive days (day 7 to day 11), after which they were euthanized on the 14th day.

**Analysis of bronchoalveolar lavage fluid (BALF).** The study involved the administration of anesthesia to mice followed by exsanguination through incising the inferior vena cava. Subsequently, bronchoalveolar lavage was conducted thrice to obtain BALFs utilizing PBS supplemented with 5 mM EDTA in 1 ml quantities. The retrieved BALFs were consolidated and subjected to centrifugation at 700 g for 5 min at 4 °C. The cellular pellets were suspended again in PBS, and the total cell count was determined using a Cellometer provided by Nexcelom. Wright-Giemsa staining was used to identify and enumerate eosinophils, neutrophils, and lymphocytes.

**Flow cytometry.** Broncho alveolar lavage (BAL) was extracted from treated mice using tracheal injection and retraction with 1 ml of saline solution (NaCl 0.9%). The collected cells were centrifuged at 350 rcf for 5 minutes, and subsequently washed twice with PBS. Prior to immunostaining, cells were treated with purified anti-CD16/CD32 antibody (Biolegend) for 10 minutes at 4°C to prevent non-specific binding. APC/Cyanine7 anti-mouse CD45 antibody (Biolegend), PerCP/Cyanine5.5 anti-mouse/human CD11b antibody (Biolegend), PE anti-mouse CD170 antibody (Siglec-F), and APC Hamster Anti-Mouse CD11c antibody (BD Pharmingen) were subsequently added, and cells were incubated for 30 minutes at 4°C in the dark. After two washes with PBS, cells were analyzed using a BD FACSCanto II flow cytometer, and data were analyzed with FlowJo software (TreeStar, Mountain View, CA).

To assess the distribution of specific immune cell subsets, a pulmonary perfusion technique was utilized. Lungs were treated with a digestion buffer consisting of RPMI 1640 supplemented with collagenase IV (1 mg/ml) and DNase I (5 U/ml) obtained from Sigma-Aldrich. Following removal from mice, lungs were sectioned into small fragments, immersed in 2 ml of the digestion buffer at a temperature of 37 °C with shaking for 1 hour. The resultant cell suspension was filtered through a 70-mm cell strainer, and the erythrocytes were eliminated following lysis with RBC lysis buffer obtained from eBioscience. The number of cells collected was determined by centrifugation and quantification. For flow cytometry analysis, cells were pre-treated with Fc-blocking antibody (BioLegend's purified anti-mouse CD16/32) in order to prevent FcγRIII/II binding. This was followed by a 1-hour incubation with fluorochrome-conjugated antibodies at 4 °C. Subsequently, samples were washed, and analysis was performed on a BD LSRII Flow Cytometer with the aid of FlowJo software (TreeStar).

**Analysis of immunoglobulin levels.** The serum levels of total IgE, HDM-specific IgE, IgG1, and IgG2a were measured using ELISA kits purchase from Wuhan Biotech Co, Ltd, China according to a standard published protocol (19).

**Lung function measurements.** Airway hyperresponsiveness was assessed through ascending concentration of methacholine (1.5625-25 mg/ml) by employing the Buxco FinePointe system, as directed by the manufacturer. The mice subjects were first rendered anesthetized by means of intra-peritoneal injection utilizing 240 mg/kg tribromoethanol (Avertin, Sigma) before being intubated with an 18-gauge catheter. The respiratory frequency of the mice was then calibrated to 140 breaths per minute with a tidal volume of 0.2 mL and a positive end-expiratory pressure of 2 mL H2O. Subsequently, the mice were administered gradually increasing amounts of aerosolized methacholine (1.5625, 3.125, 6.25, 12.5, and 25 mg/ml), and resistance was determined and recorded with a plethysmograph (Buxco). Finally, the baseline resistance was restored before administration of subsequent doses of methacholine.

**Histology.** Excised lungs were fixed in 4% paraformaldehyde, sectioned and stained with H&E or periodic acid-Schiff (PAS). The samples were examined for inflammatory cell infiltration, tissue damage, and mucus production using a Nikon E200 microscope.

**Cytokine production.** Broncho alveolar lavage (BAL) fluid of treated mice was harvested. After centrifugation at 350 rcf for 5 min, supernatant was collected. Levels of mouse IgE, IL-5 and IL-13 were assessed by ELISA according to the manufacturer’s protocol (4A Biotech, Beijing, China).

Single-cell suspensions were obtained by homogenizing mediastinal lymph nodes through a 100-µm cell sieve. Cells were treated ex vivo with 15 µg/ml HDM for 3 days, and supernatants were analyzed using ELISA kits to detect IL-5, IL-13, IL-17A, IL-10, and IFN-γ (Shanghai MLBIO Biotechnology Co. Ltd).

**Preparation of lung homogenates.** The lung of mouse were homogenized in 500 µl cold lysis buffer (20). Samples were kept on ice for 30 min and centrifuged to discard cellular debris. Protein contents were measured on the supernatants.

**Epithelial cell sorting.** The present study describes the methodology used to isolate and analyze lung cells. The lungs were subjected to digestion with Collagenase and Dnase I at 37°C for 1.5 hours. The resulting cells were stained with antibodies against CD45, EpCam, Sca-1, and CD31 for 30 minutes at 4°C, with exclusion of dead cells using DAPI staining. Subsequently, a FACS Aria III (BD) was used to sort the cells, which were then stored at -20°C in TriPure isolation reagent (Roche). RNA was extracted from the sorted cells using chloroform/isopropanol, followed by reverse transcription using the Transcriptor High Fidelity cDNA Synthesis kit (Roche). The obtained cDNA was then subjected to real-time quantitative PCR using a two times master mix (Bioline) on a LCII 480 (Roche), and the expression levels were normalized to the hypoxanthine-guanine phosphoribosyl transferase (HPRT) expression. These results provide valuable insight into the molecular mechanisms underlying lung development and function.

**Transcriptomic Analysis.** Total RNA samples were isolated from mouse lung tissues using a commercially available mirVanaTM miRNA isolation kit (Ambion, Invitrogen, Carlsbad, CA). Poly-T oligo-attached magnetic beads were used to purify mRNA from the total RNA samples, which were then randomly fragmented in a fragmentation buffer for the construction of sequencing libraries. TruSeq Stranded mRNA LT Sample Prep Kit (Illumina) was used to prepare the sequencing libraries, and the libraries were sequenced using an Illumina HiSeqTM 2500 instrument to generate 150-bp paired-end reads. The initial sequencing data was processed by filtering out low-quality reads and reads containing adaptors to obtain clean reads. The processed reads were subsequently mapped to a reference genome utilizing HISAT2, and gene expression levels were quantified using HTSeq (version 0.9.1) by calculating fragments per kilobase of transcript per million mapped reads. DESeq2 (version 1.18.0) was utilized to conduct differential gene expression analysis, and genes demonstrating significant differences (*p* < 0.05) were designated as differentially expressed genes (DEGs). Cluster analysis was applied to the DEGs to identify gene expression patterns, and Gene Ontology (GO) enrichment analysis of DEGs was performed using GOseq R packages. KEGG Orthology-Based Annotation System (KOBAS) was employed to test for significant enrichment of DEGs in KEGG pathways. Additionally, Gene Set Enrichment Analysis (GSEA) was conducted to determine the enrichment pathways generated from the two DEG sets described above, using a stringent cutoff of FDR < 0.25 and P < 0.05.

To establish the interrelationship between transcript levels and treatment group (control vs treatment) through the application of Weighted Gene Co-Expression Network Analysis (WGCNA) technique available in R package (21). Initially, to build gene networks utilizing a scale-free topology model, we computed expression correlation coefficients and selected an appropriate soft threshold. Thereafter, we employed the dynamic tree cut method (minModuleSize = 50 and mergeCutHeight = 0.25) to identify gene expression modules displaying similar patterns, based on a gene cluster dendrogram. Subsequently, we calculated module eigengenes to identify modules significantly linked to the traits of samples, which were subsequently correlated with the groups. We considered the modules that demonstrated high correlation values (P < 0.05), as significant trait-related modules. Moreover, we identified putatively related genes to the group based on their correlation values (P < 0.05).

**Validation of transcriptome analysis using quantitative real time PCR (RT-PCR).** RNA from lungs was purified using an RNA isolation kit (Tiangen) and then reversely transcribed (Tiangen). cDNA was analyzed by qPCR using SYBR green reagents (Thermo) with the ABI 7300 system. The primers used for RT-PCR were listed in Table S5. GAPDH was used as the reference gene to normalize the gene expression data.


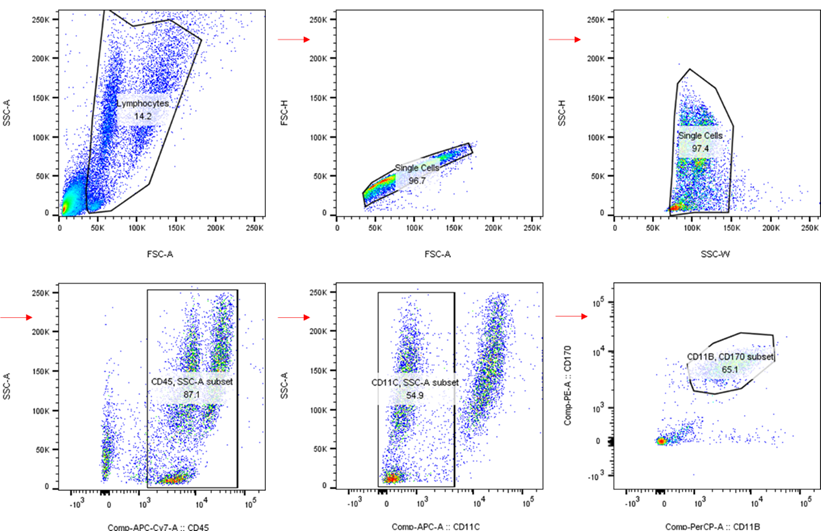


**Figure S1.** Gating strategy of the essential cell subsets in the asthma mouse model for flow cytometry data analysis. The strategy followed to gate FSC-A x SSC-A -> FSC-A x FSC-H -> SSC-W x SSC-H-> CD45+ -> CD11b--> CD170+CD11b+ from flow cytometry data analysis is shown by the red arrows.


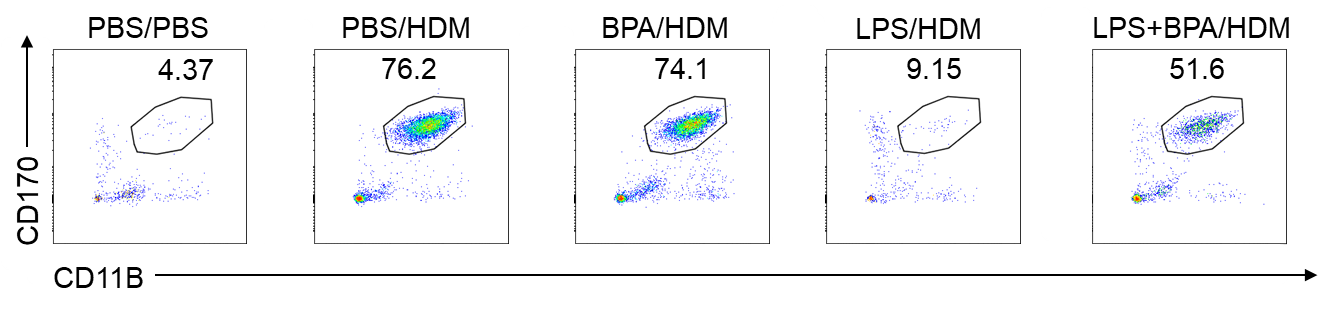


**Figure S2.** Cyto-flow analysis of CD11B and CD170 subsets in BALF.


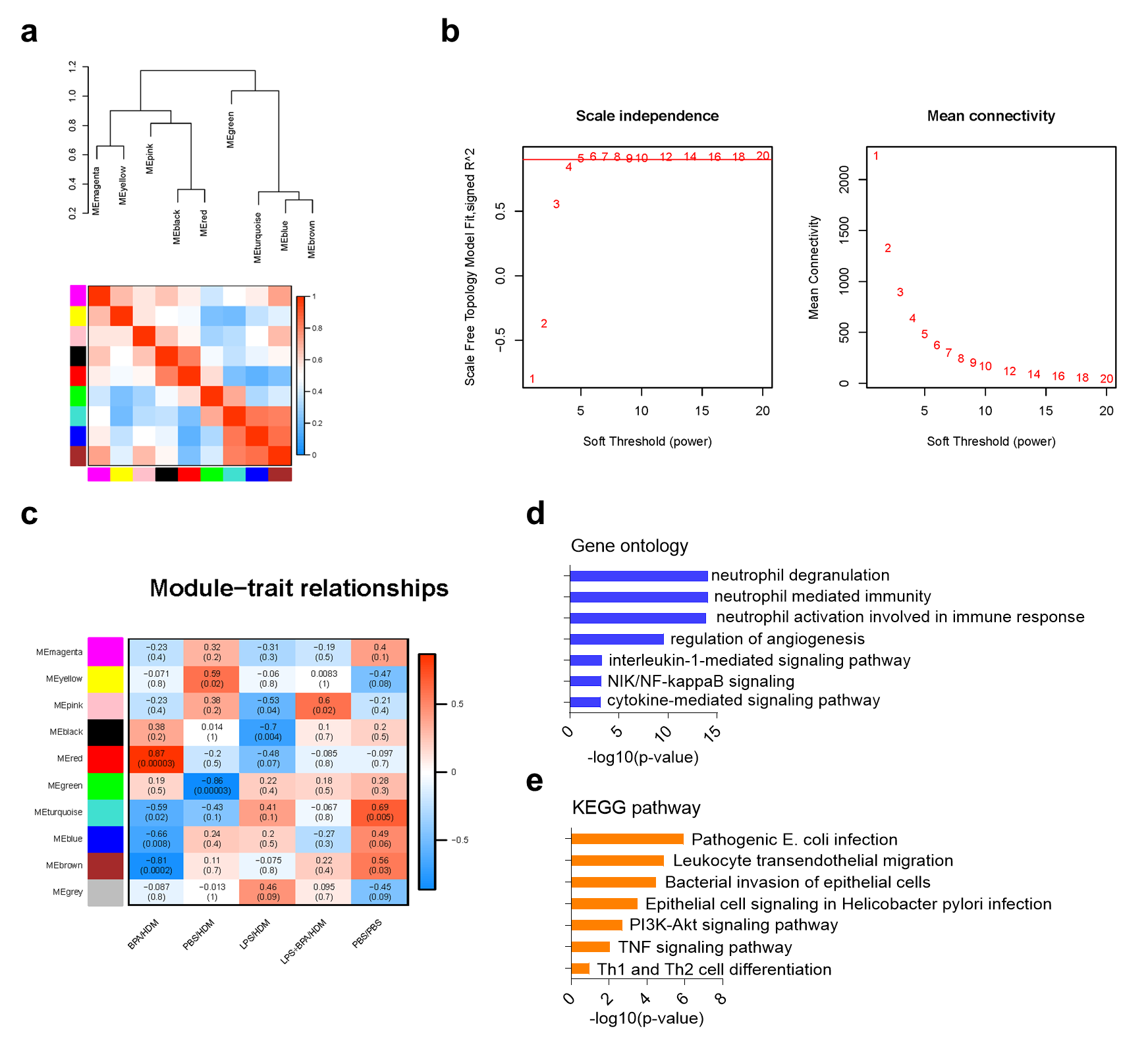


**Figure S3. Weighted Gene Coexpression Network Analysis (WGCNA). a** Unsupervised hierarchical clustering heatmap and dendrogram of module eigengenes. **b** Scale independence and mean connectivity analysis. **c** Module-trait relationships. **d** GO enrichment analysis of Black modules. **e** KEGG enrichment analysis of Black modules.

**Figure S4.** RT-PCR results of 4 selected genes (IL-5, IL-10, IFN-γ, and Tnfaip3) in mouse lung tissues from different treated groups. Data are presented as the mean ± SEM of triplicates compared with the control PBS group. **p* < 0.05, ***p* < 0.01 (ANOVA).

**Figure S5.** LAL activity of the FPLC-purified LPS/BPA complex and the unfractionated LPS/BPA mixture (1 mM BPA and 100 ng/ml LPS). Values represent the mean and standard error relative to the control (fractionated pure LPS), which was prepared using the identical conditions as those used to purify the LPS/BPA complex. n.s., not significant (ANOVA).


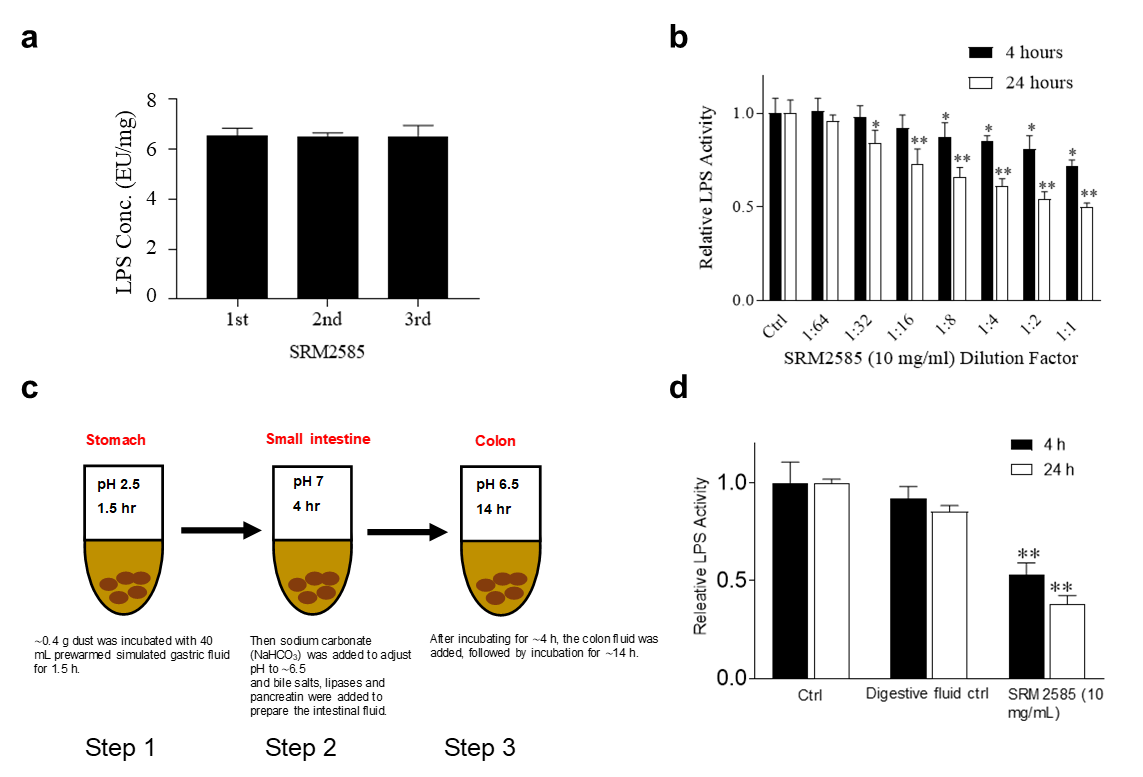


**Figure S6.** **LPS activity neutralization using SRM2585.** **a** LPS levels in a dust Standard Reference Material [SRM 2585, Organic Contaminants in House Dust; National Institute of Standards and Technology (NIST), Gaithersburg, MD]. Values are presented the average and standard error of duplicate analysis of three independent experiments. **b** Dose response of LPS activity neutralization using SRM2585 during 4 and 24 hours’ incubation. **c** Flow chart displaying the steps involving biological extraction of dust. **d** Extracts from SRM2585 (10 mg/ml) using simulated digestive fluids neutralize LPS activity after incubation for 4 h and 24 h. Values are means and the standard error relative to the LPS control (n = 3, 30000 EU/ml). Signiﬁcant differences versus control are indicated as **p* < 0.05 and ***p* < 0.01 (*ANOVA*).


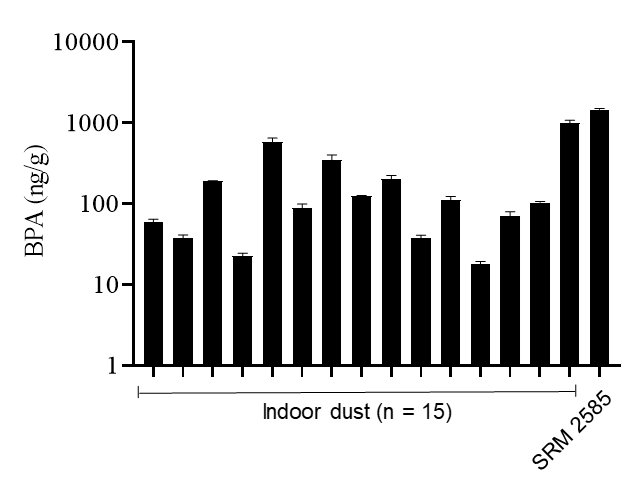


**Figure S7.** BPA concentrations (ng/g) in indoor dust samples (n=15) and one dust standard reference material (SRM2585). The bar represents the average and standard error of duplicate analysis.


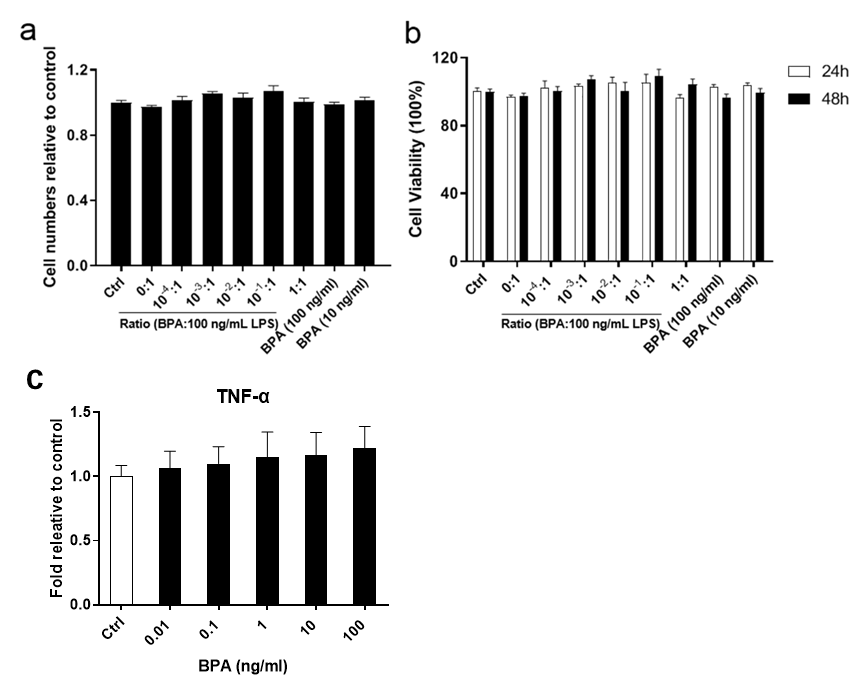


**Figure S8. a** Relative cell number change of THP-1 macrophages exposed to either BPA, LPS, or co-exposure to BPA with LPS at 24 h after exposure. **b** Viability of THP-1 cells exposed to BPA, LPS, or BPA + LPS. Cells were analysed 24 h and 48 h after exposure. **c** Bisphenol A alone without LPS did not affect the pro-inflammatory cytokine release in THP-1 macrophages. Values are means and the standard error of means relative to the 0.1% DMSO vehicle control (n = 3).


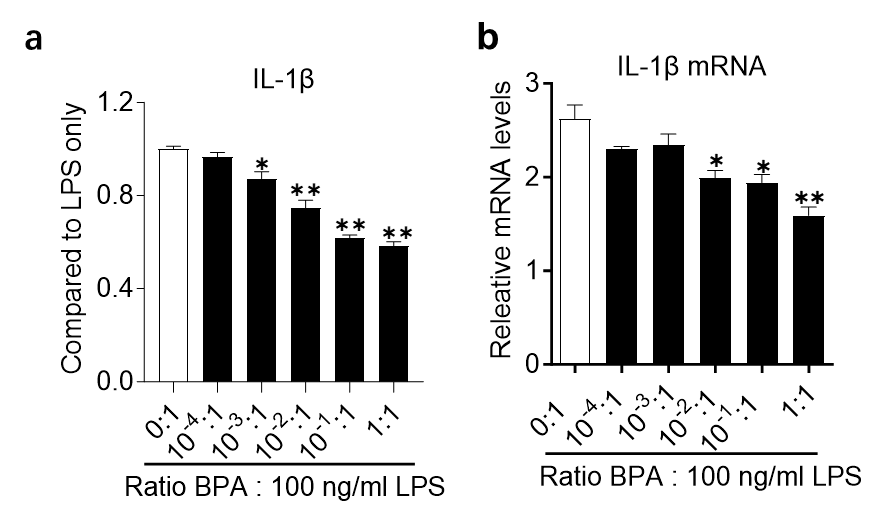


**Figure S9. a** Inhibition of LPS-induced production of IL-1β in THP-1 cells by BPA at various concentrations (ng/ml). **b** The effects of BPA on IL-1β mRNA expression in THP-1 cells.


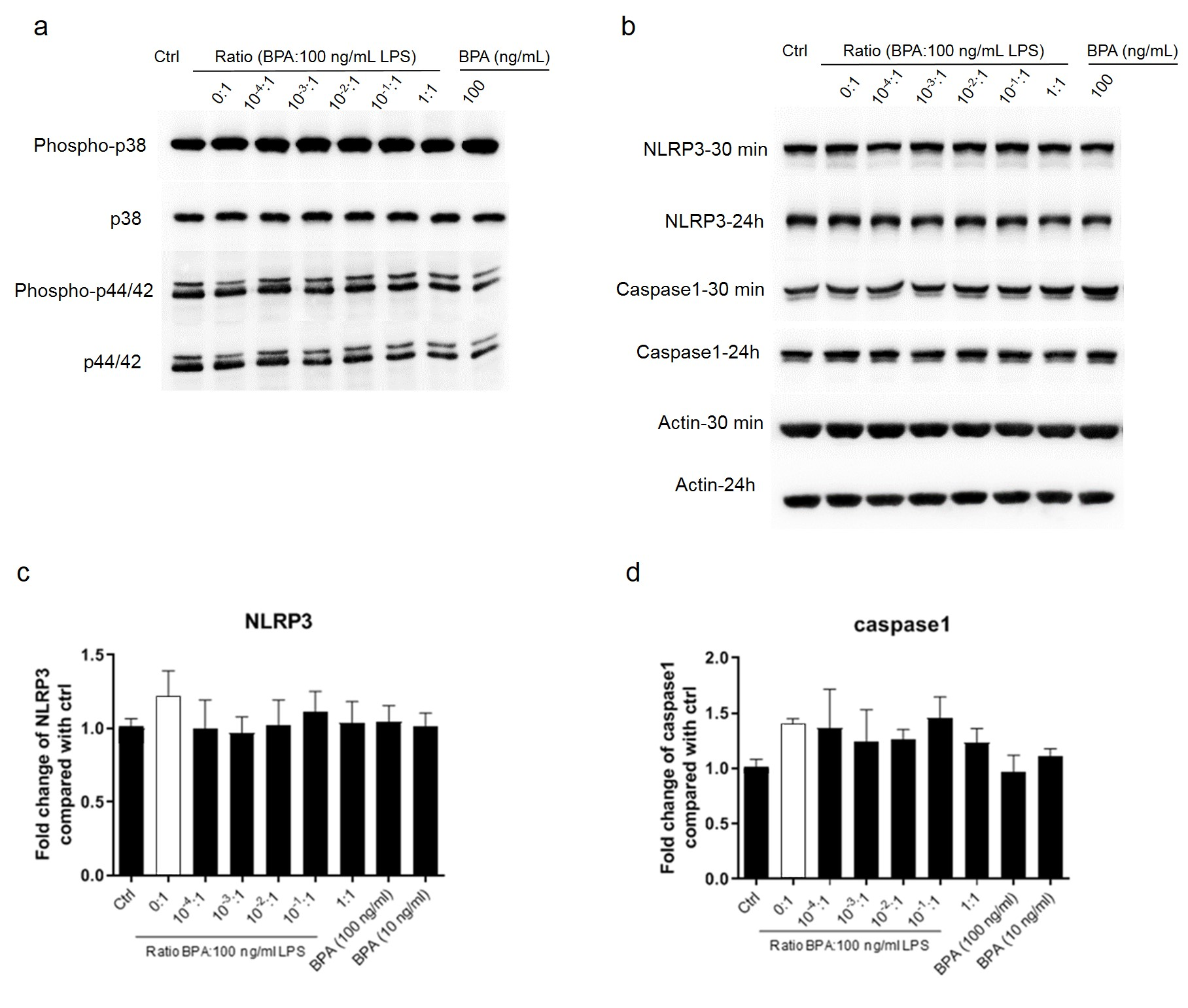


**Figure S10. a** Western blot analysis of the phosphorylation of p38 and p44/42 in THP-1 macrophages co-treated with LPS and BPA for 30 min; **b** Western blot analysis of NLRP3, caspase 1, and actin (loading control) for 30 min and 24 h and **c, d** qPCR analysis of NLRP3 **c** and caspase1 mRNA **d** level in THP-1 macrophages co-treated with LPS and BPA for 24 h. Values are means and the standard error of means relative to the 0.1% DMSO vehicle control (n = 3).

**Figure S11.** Effect of preincubating LPS with BPA for 0, 2 and 12 h on TNF-α production by THP-1 cells. Values represent the mean and standard error relative to the LPS-alone control at each time (n = 3). Significant differences vs. the control: ***p* < 0.01.


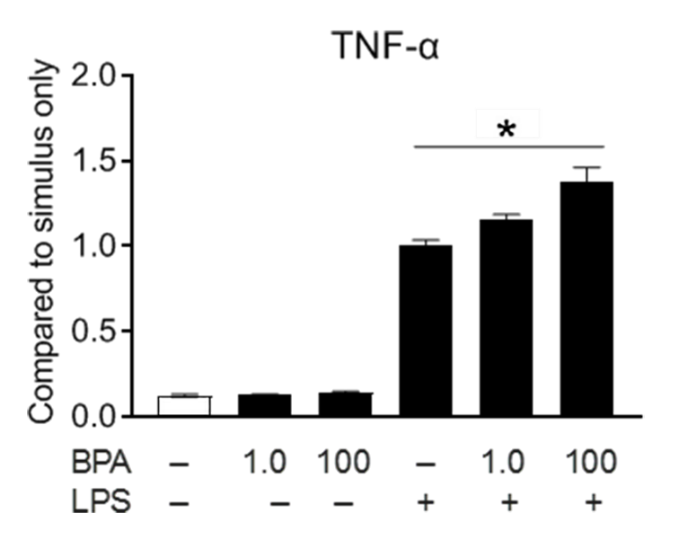


**Figure S12**. Effect of BPA on TNF-α production after treating THP-1 cells with 1.0 ng/ml or 100 ng/ml BPA for 6 h, washing the cells with medium, and then treating them with 100 ng/ml LPS for 2 h. The blank control group was cultured in BPA-free medium for 8 h, and the LPS control group was cultured for 6 h in BPA-free medium and then treated with LPS for 2 h. The BPA control group was simultaneously incubated for 2 h. Values represent the mean and standard error of the mean vs. cells treated with 0.1% DMSO (vehicle) simultaneously treated with *E. coli* LPS (n = 3). Significant differences vs. the control co-treated with *E. coli* LPS: **p* < 0.05.


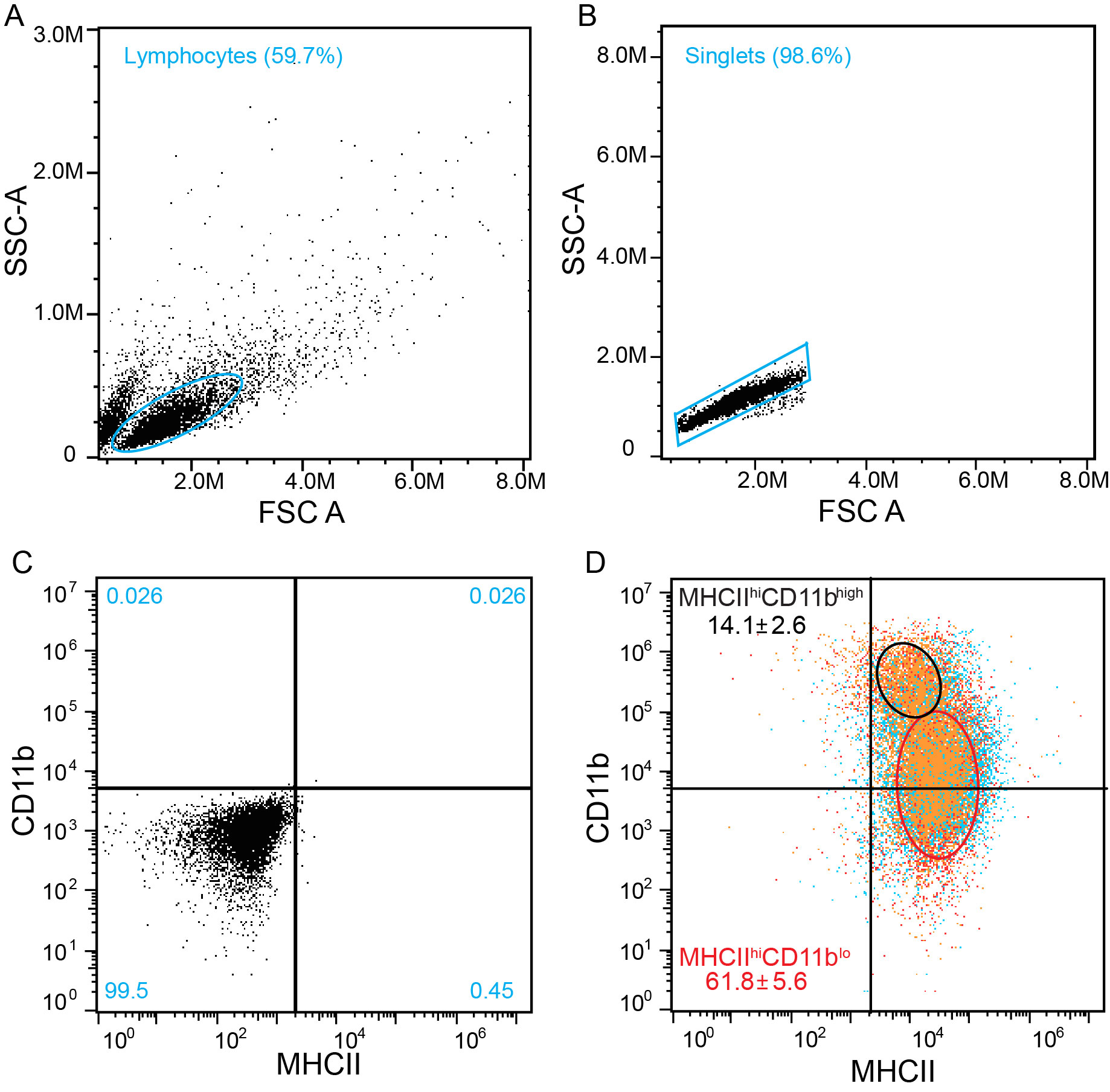


**Figure S13.** **a** General gating strategies to identify mature DCs from cultured BMDCs. **b** Lymphocytes are detected via side-scatter area (SSC-A) and forward scatter area (FSC-A), where they are further gated for singlets. **c** Unstained cells gated for CD11b and MHCII. **d** Mature DCs expressing cell surface marker CD11b and MHCII, making up approximately 80% of total cells and confirming phenotypical and functional maturation of DCs.


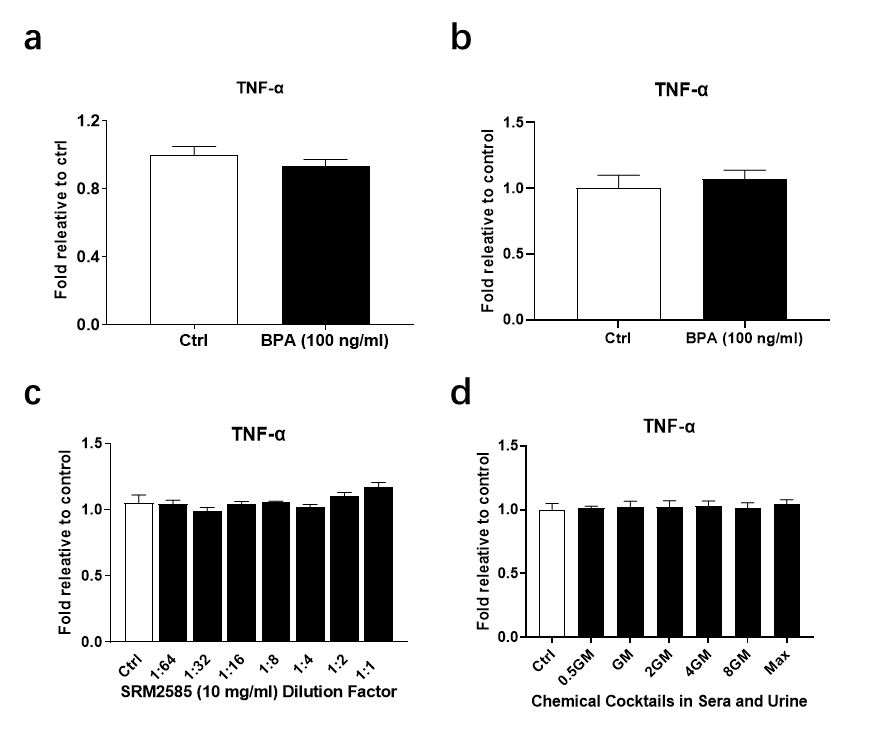


**Figure S14.** Effects of Bisphenol A (BPA), SRM2585, and a chemical cocktail on pro-inflammatory cytokine release in immune cells. **a** BPA at the high dose without lipopolysaccharide (LPS) did not affect the release of tumor necrosis factor alpha (TNF-α) from human alveolar macrophages. **b** BPA at the high dose without LPS did not affect the release of TNF-α from mouse bone marrow-derived dendritic cells (BMDCs). **c** SRM2585 alone without LPS-treatment did not induce pro-inflammatory cytokine release in THP-1 cells. **d** A chemical cocktail at human-relevant concentration alone without LPS co-treatment did not induce pro-inflammatory cytokine release in THP-1 cells compared with the 0.1% dimethyl sulfoxide (DMSO) vehicle control. GM is Geometric Mean. Values are means and the standard error of means relative to the 0.1% DMSO vehicle control (n = 3).

**Figure S15.** Bioactivity of LPS from *E. coli* O55:B5 and *E. coli* O111:B4 at the concentration of 100 ng/ml. Data presented as mean ± SEM of triplicates compared with LPS from *E. coli* O55:B5.

**Table S1.** Chemical names, acronym, CAS number, LPS release reduction and LPS bioactivity neutralization potency (EC_10/50_) and efficacy (Max) results for 17 tested compounds

|  | | Reduction | | | | | Neutralization | | | | |
| --- | --- | --- | --- | --- | --- | --- | --- | --- | --- | --- | --- |
| Compound name  (Acronym) | CAS  Number | LR  Max^a^ | EC_10_ | | EC_50_ (μM) | | LN  Max^b^ | EC_10_ | | EC_50_ | |
|  |  |  | μM | ng/ml | μM | ng/ml |  | μM | ng/ml | μM | ng/ml |
| Tetrabromo bisphenol A  (TBBPA) | 79-94-7 | 0.65 | < 0.1 | <54.39 | 60.7 | 33,014 | 0.87 | < 0.1 | <54.39 | 4.5 | 2,448 |
| Tris(1,3-dichloro-2-propyl)phosphate  (TDCPP) | 237-159-2 | 0.82 | < 0.1 | <43.09 | 35.5 | 15,297 | 0.53 | < 0.1 | <43.09 | 651 | 280,509 |
| Triphenyl phosphate  (TPP) | 115-86-6 | 0.64 | < 0.1 | <32.62 | 62.7 | 20,458 | 0.69 | < 0.1 | <32.63 | 459 | 149,762 |
| Bisphenol A diglycidyl ether  (BADGE) | 1675-54-3 | 0.83 | < 0.1 | <34.05 | 29.7 | 10,113 | 0.63 | < 0.1 | <34.05 | 15.4 | 5,243 |
| Bisphenol A  (BPA) | 80-05-7 | 0.96 | < 0.1 | <22.83 | 3.20 | 731 | 0.96 | < 0.1 | <22.83 | 2.29 | 523 |
| Benzyl Butyl Phthalate  (BBP) | 85-68-7 | 0.69 | 0.13 | 40.61 | 50.8 | 15,868 | 0.87 | < 0.1 | <31.24 | 4.51 | 1,409 |
| Bis(2-ethylhexyl) phthalate  (DEHP) | 117-81-7 | 0.24 | 6.92 | 2,703 | N/A | N/A | 0.17 | 0.10 | 39.06 | N/A | N/A |
| Bisphenol S  (BPS) | 80-09-1 | 0.25 | 33.1 | 8,284 | N/A | N/A | 0.41 | 2.40 | 600 | N/A | N/A |
| Methyl paraben  (MP) | 99-76-3 | 0.62 | < 0.1 | <15.22 | 65.3 | 9,935 | 0.62 | 4.37 | 665 | 221 | 33,625 |
| Butyl paraben  (BP) | 94-26-8 | 0.97 | < 0.1 | <19.42 | 63.6 | 12,353 | 0.97 | 0.19 | 36.9 | 117 | 22,647 |
| Ethyl paraben  (EP) | 120-47-8 | 0.68 | 4.11 | 683 | 794 | 131,942 | 0.48 | 7.13 | 1,184 | N/A | N/A |
| Sodium benzoate  (SB) | 532-32-1 | 0.56 | 100 | 14,411 | 2368 | 341,252 | 0.63 | 37.4 | 5,390 | 2,362 | 340,387 |
| Benzoic acid  (BA) | 65-85-0 | 0.20 | 3981 | 486160 | N/A | N/A | 0.47 | 83.2 | 10,160 | N/A | N/A |
| Triclosan  (TCS) | 3380-34-5 | 0.37 | < 0.1 | <28.95 | N/A | N/A | 0.01 | N/A | N/A | N/A | N/A |
| Ascorbic acid  (AA) | 50-81-7 | 0.30 | 2.67 | 470 | N/A | N/A | 0.42 | 806.8 | 142,094 | N/A | N/A |
| Nonylphenol  (NP) | 104-40-5 | 0.80 | < 0.1 | <22.04 | 21.3 | 4,693 | 0.56 | 3.02 | 665 | 39.81 | 8,772 |
| Ethanol  (EtOH) | 64-17-5 | N/A | N/A | N/A | N/A | N/A | 0.01 | N/A | N/A | N/A | N/A |

^a^LPS release reduction (LR) (relative to vehicle control) and ^b^LPS bioactivity neutralization (LN) (relative to vehicle control) provided as maximum efficacies. EC_10_ and EC_50_ values in μM and ng/ml (concentration required to reduce effect by 10% and 50%; respectively). NA, no effect higher than 10% or 50% neutralization at the tested dosing.

**Table S2.** Literature summary of tested indoor chemicals and their reported effect on human immune disorders

| **Chemicals** | **Human immune disorders** |
| --- | --- |
| Tetrabromo bisphenol A | Aggravation of immune/allergic responses (22) and Pneumonia (23) |
| Tris(1,3-dichloro-2-propyl)phosphate | Thyroid autoimmunity (24), perturbation of immune function (25), and immunotoxicity (26) |
| Triphenyl phosphate | Immunotoxicity (27, 28) |
| Bisphenol A | Neurological autoimmune disorders (29) and thyroid autoimmunity (30) |
| Phthalate | Systemic lupus erythematosus (31) and testicular autoimmunity (32, 33) |
| Bisphenol S | Immunotoxicity (34) |
| Triclosan and paraben | Allergen sensitization and wheeze (35, 36) |
| Benzoic acid, sodium benzoate and ascorbic Acid | Immunomodulatory effects (37, 38) |
| Nonylphenol | Inflammatory bowel disease (39) and increase of autoimmune susceptibility and/or exacerbate existing autoimmune conditions (40) |
| Ethanol | Immunodeficiency (41, 42) |

**Table S3.** The maximum, geometric mean urinary or blood concentrations in human biological samples for the selected chemicals with the defined sample sizes.

|  | Urine or blood concentrations (ng/ml) | | | |
| --- | --- | --- | --- | --- |
| Chemical | Maximum | Geometric mean | Sample Size (n) | References |
| TBBPA | 7.6 | 0.7 | 265 | (43-45) |
| TDCPP | 8.6 | 0.9 | 2,646 | (46) |
| TPP | 16.6 | 2.7 | 69 | (47-49) |
| BADGE | 2.5 | 0.5 | 223 | (50) |
| BPA | 12.9 | 1.9 | 15,593 | (46) |
| BBP | 232.8 | 16.7 | 18,462 | (46) |
| DEHP | 100.0 | 0.163 | 134 | (51) |
| BPS | 4.7 | 0.4 | 2,682 | (46) |
| MP | 1,056.6 | 52.4 | 13,076 | (46) |
| BP | 18.28 | 1.2 | 13,076 | (46) |
| EP | 99.6 | 5.4 | 13,076 | (46) |
| TCS | 24.5 | 13.8 | 15,593 | (46) |
| NP | 1.57 | < 0.1 | 394 | (52) |

**Table 4.** The primer sequences of target mRNA genes.

| **Gene** | **Sequence** |
| --- | --- |
| Il5 | F: 5' ATGAAGTGCTGGAGATGG 3'  R: 5' GGATGCTAAGGTTGGGTATG 3' |
| Il10 | F: 5' AGGACTTTAAGGGTTACTTG 3'  R: 5' CAAATGCTCCTTGATTTCTG 3' |
| CCL20 | F: 5' TCC AGA GCT ATT GTG GGT TTCA-3'  R: 5' CTG AGG AGG TTC ACA GCC CTT-3' |
| GM-CSF | F: 5' TCA AAG AAG CCC TGA ACC TC-3'  R: 5' AAA TTG CCC CGT AGA CCC TGCT-3' |
| IL-33 | F: 5' ACT ATG AGT CTC CCT GTC CTG-3'  R: 5' ACG TCA CCC CTT TGA AGC-3' |
| Ifnγ | F: 5' TCAAGTGGCATAGATGTG 3'  R: 5' TGTTGCTGAAGAAGGTAG 3' |
| Tnfaip3 | F: 5' AGTATCCCTGCCTCCTGTC 3'  R: 5' TGCTTGTCCCTGCTCTGTC 3' |
| Gapdh | F: 5' ATCACTGCCACCCAGAAG 3'  R: 5' TCCACGACGGACACATTG 3' |

**Table S5.** Expression of direct target genes of estrogen receptors as previously identified by Lin et al (53).

|  | BPA/HDM  VS PBS/HDM | | LPS:BPA/HDM  VS LPS/HDM | |  | BPA/HDM  VS PBS/HDM | | LPS:BPA/HDM  VS LPS/HDM | |
| --- | --- | --- | --- | --- | --- | --- | --- | --- | --- |
|  | fold change | *P* value | fold change | *P* value |  | fold change | *P* value | fold change | *P* value |
| Esr1 | 1.33 | 0.24 | 1.28 | 0.20 | Nop16 | 1.05 | 0.52 | 0.95 | 0.12 |
| Esr2 | 0.91 | 0.52 | 1.12 | 0.56 | Spg21 | 0.97 | 0.53 | 1.01 | 0.78 |
| Hmgcr | 0.95 | 0.49 | 1.15 | 0.14 | Bri3bp | 0.84 | 0.02 | 1.06 | 0.63 |
| Thbs1 | 0.89 | 0.61 | 0.99 | 0.96 | Lor | 1.62 | 0.23 | 1.15 | 0.46 |
| Rapgefl1 | 0.74 | 0.15 | 0.84 | 0.21 | Mpp3 | 0.75 | 0.31 | 0.98 | 0.89 |
| Olfm1 | 0.78 | 0.02 | 1.05 | 0.28 | Jak1 | 0.89 | 0.07 | 1.15 | 0.01 |
| Dnajc1 | 1.01 | 0.61 | 0.98 | 0.70 | NOL5A | 1.04 | 0.45 | 0.94 | 0.39 |
| Igfbp4 | 0.66 | 0.09 | 1.34 | 0.13 | Afg3l2 | 1.05 | 0.08 | 1.00 | 0.93 |
| Abca3 | 0.90 | 0.07 | 1.08 | 0.39 | Cish | 0.95 | 0.55 | 0.98 | 0.89 |
| Stc2 | 1.07 | 0.78 | 0.84 | 0.23 | Padi3 | 1.20 | 0.57 | 0.52 | 0.12 |
| Peak1 | 0.98 | 0.71 | 0.93 | 0.25 | Uggt1 | 0.87 | 0.04 | 1.08 | 0.38 |
| Slc38a1 | 0.87 | 0.52 | 0.90 | 0.45 | Vps13c | 0.90 | 0.13 | 1.09 | 0.15 |
| Atp13a3 | 0.85 | 0.06 | 0.99 | 0.85 | Adcy9 | 0.93 | 0.05 | 1.00 | 0.97 |
| Elovl2 | 0.46 | 0.19 | 2.97 | 0.36 | Ahcyl1 | 1.00 | 0.96 | 1.00 | 0.91 |
| Hspb8 | 1.23 | 0.07 | 1.17 | 0.10 | Xkr8 | 1.03 | 0.74 | 0.97 | 0.77 |
| Greb1 | 0.94 | 0.18 | 0.92 | 0.56 | Bambi | 1.27 | 0.02 | 1.02 | 0.84 |
| Rbbp8 | 0.91 | 0.16 | 0.95 | 0.37 | Ndrg1 | 0.91 | 0.26 | 1.17 | 0.07 |
| Siah2 | 1.14 | 0.18 | 0.86 | 0.25 | Arid5b | 1.04 | 0.52 | 1.02 | 0.28 |
| Cxcl12 | 0.82 | 0.13 | 1.00 | 0.97 | Nfia | 1.16 | 0.12 | 0.87 | 0.15 |
| Fzd8 | 1.12 | 0.22 | 0.91 | 0.48 | Serpine1 | 1.00 | 0.99 | 1.25 | 0.28 |
| Dnajc12 | 1.48 | 0.14 | 0.73 | 0.03 | Epha4 | 1.02 | 0.80 | 1.48 | 0.02 |
| Nif3l1 | 1.00 | 0.98 | 0.97 | 0.68 | Cdc42ep3 | 1.08 | 0.19 | 0.91 | 0.34 |
| Thsd4 | 1.22 | 0.19 | 0.97 | 0.79 | Kcng1 | 0.93 | 0.78 | 0.87 | 0.59 |
| Zmiz1 | 0.89 | 0.16 | 1.16 | 0.25 | St6gal1 | 1.09 | 0.25 | 0.97 | 0.71 |
| Scn1b | 1.19 | 0.17 | 0.96 | 0.76 | Epha4 | 1.02 | 0.80 | 1.28 | 0.32 |
| Tpd52l1 | 1.15 | 0.34 | 0.89 | 0.62 | F10 | 0.45 | 0.07 | 1.27 | 0.22 |
| Dgkz | 0.97 | 0.64 | 1.04 | 0.36 | Alox12b | 0.72 | 0.43 | 0.72 | 0.65 |
| Cadm1 | 0.98 | 0.80 | 1.14 | 0.57 | Ccng2 | 1.15 | 0.05 | 1.06 | 0.24 |
| Arpp21 | 0.12 | 0.06 | 1.00 | 1.00 | Cd7 | 0.95 | 0.73 | 1.14 | 0.54 |
| Cycs | 1.09 | 0.42 | 1.14 | 0.36 | Phb2 | 1.00 | 0.93 | 0.95 | 0.15 |
| Pgr | 0.96 | 0.72 | 1.07 | 0.79 | Lmcd1 | 1.02 | 0.88 | 0.90 | 0.40 |
| Amd1 | 0.96 | 0.69 | 1.01 | 0.88 | Mknk2 | 0.95 | 0.35 | 1.13 | 0.16 |
| Il6st | 0.99 | 0.87 | 1.11 | 0.34 | Crabp2 | 4.25 | 0.24 | 0.33 | 0.38 |
| Nrip1 | 0.82 | 0.08 | 1.16 | 0.12 | Ctbs | 0.91 | 0.17 | 1.14 | 0.23 |
| Ptges | 0.91 | 0.25 | 1.04 | 0.68 | Plk2 | 1.08 | 0.43 | 1.01 | 0.96 |
| Ahsa1 | 0.96 | 0.57 | 0.90 | 0.28 | Pxylp1 | 1.36 | 0.08 | 1.07 | 0.66 |
| Azin1 | 0.96 | 0.34 | 1.06 | 0.44 | Pafah1b1 | 0.94 | 0.26 | 1.08 | 0.08 |
| Ppp1r15b | 0.93 | 0.06 | 1.06 | 0.17 | Hilpda | 1.23 | 0.06 | 1.23 | 0.31 |
| Ctsd | 0.79 | 0.02 | 1.13 | 0.13 | Agap2 | 0.87 | 0.22 | 1.10 | 0.62 |
| Galnt4 | 0.95 | 0.45 | 1.07 | 0.23 | Bmp7 | 1.10 | 0.73 | 0.99 | 0.95 |
| Cep350 | 0.94 | 0.05 | 1.06 | 0.17 |  |  |  |  |  |

**Supplementary References**

1. R. A. Rudel, D. E. Camann, J. D. Spengler, L. R. Korn, J. G. Brody, Phthalates, alkylphenols, pesticides, polybrominated diphenyl ethers, and other endocrine-disrupting compounds in indoor air and dust. *Environ Sci Technol.* **37**, 4543-4553 (2003).

2. H. M. Stapleton *et al.*, Determination of polybrominated diphenyl ethers in indoor dust standard reference materials. *Anal Bioanal Chem.* **384**, 791-800 (2006).

3. C. D. Kassotis, K. Hoffman, H. M. Stapleton, Characterization of Adipogenic Activity of House Dust Extracts and Semi-Volatile Indoor Contaminants in 3T3-L1 Cells. *Environ Sci Technol.* **51**, 8735-8745 (2017).

4. M. Fang, H. M. Stapleton, Evaluating the bioaccessibility of flame retardants in house dust using an in vitro Tenax bead-assisted sorptive physiologically based method. *Environ Sci Technol.* **48**, 13323-13330 (2014).

5. M. Fang, T. F. Webster, H. M. Stapleton, Activation of Human Peroxisome Proliferator-Activated Nuclear Receptors (PPARgamma1) by Semi-Volatile Compounds (SVOCs) and Chemical Mixtures in Indoor Dust. *Environ Sci Technol.* **49**, 10057-10064 (2015).

6. M. Fang, T. F. Webster, P. L. Ferguson, H. M. Stapleton, Characterizing the peroxisome proliferator-activated receptor (PPARgamma) ligand binding potential of several major flame retardants, their metabolites, and chemical mixtures in house dust. *Environ Health Perspect.* **123**, 166-172 (2015).

7. A. G. Oomen *et al.*, Comparison of five in vitro digestion models to study the bioaccessibility of soil contaminants. *Environ Sci Technol.* **36**, 3326-3334 (2002).

8. M. Fang, H. M. Stapleton, Evaluating the bioaccessibility of flame retardants in house dust using an in vitro Tenax bead-assisted sorptive physiologically based method. *Environ Sci Technol.* **48**, 13323-13330 (2014).

9. P. M. Jones, M. J. Bennett, Clinical applications of 3-hydroxy fatty acid analysis by gas chromatography-mass spectrometry. *Biochim Biophys Acta.* **1811**, 657-662 (2011).

10. P. M. Jones, M. J. Bennett, 3-Hydroxy-fatty acid analysis by gas chromatography-mass spectrometry. *Methods Mol Biol.* **603**, 229-243 (2010).

11. F. C. Bernstein *et al.*, The Protein Data Bank: A computer‐based archival file for macromolecular structures. *Eur J Biochem.* **80**, 319-324 (1977).

12. X. Tang *et al.*, Cryo-EM structures of lipopolysaccharide transporter LptB(2)FGC in lipopolysaccharide or AMP-PNP-bound states reveal its transport mechanism. *Nat Commun.* **10**, 4175 (2019).

13. W. L. Jorgensen, D. S. Maxwell, J. Tirado-Rives, Development and testing of the OPLS all-atom force field on conformational energetics and properties of organic liquids. *J Am Chem Soc.* **118**, 11225-11236 (1996).

14. A. Bax, S. Grzesiek, Methodological Advances in Protein Nmr. *Acc Chem Res.* **26**, 131-138 (1993).

15. F. Delaglio *et al.*, NMRPipe: a multidimensional spectral processing system based on UNIX pipes. *J Biomol NMR.* **6**, 277-293 (1995).

16. M. B. Lutz *et al.*, An advanced culture method for generating large quantities of highly pure dendritic cells from mouse bone marrow. *J Immunol Methods.* **223**, 77-92 (1999).

17. M. F. Manzoor *et al.*, An insight into bisphenol A, food exposure and its adverse effects on health: A review. *Front Nutr.* **9**, 1047827 (2022).

18. K. B. Delclos *et al.*, Toxicity evaluation of bisphenol A administered by gavage to Sprague Dawley rats from gestation day 6 through postnatal day 90. *Toxicol Sci.* **139**, 174-197 (2014).

19. T. Polte *et al.*, CD137-mediated immunotherapy for allergic asthma. *J Clin Invest.* **116**, 1025-1036 (2006).

20. M. J. Schuijs *et al.*, Farm dust and endotoxin protect against allergy through A20 induction in lung epithelial cells. *Science* **349**, 1106-1110 (2015).

21. P. Langfelder, S. Horvath, WGCNA: an R package for weighted correlation network analysis. *BMC Bioinf.* **9**, 559 (2008).

22. E. Koike, R. Yanagisawa, H. Takigami, H. Takano, Brominated flame retardants stimulate mouse immune cells in vitro. *J Appl Toxicol.* **33**, 1451-1459 (2013).

23. W. Watanabe *et al.*, Effects of tetrabromobisphenol A, a brominated flame retardant, on the immune response to respiratory syncytial virus infection in mice. *Int Immunopharmacol.* **10**, 393-397 (2010).

24. Q. Wang *et al.*, Exposure of zebrafish embryos/larvae to TDCPP alters concentrations of thyroid hormones and transcriptions of genes involved in the hypothalamic–pituitary–thyroid axis. *Aquat Toxicol.* **126**, 207-213 (2013).

25. A. Farhat *et al.*, Tris (1, 3-dichloro-2-propyl) phosphate perturbs the expression of genes involved in immune response and lipid and steroid metabolism in chicken embryos. *Toxicol Appl Pharmacol.* **275**, 104-112 (2014).

26. D. Canbaz, A. Logiantara, R. van Ree, L. S. van Rijt, Immunotoxicity of organophosphate flame retardants TPHP and TDCIPP on murine dendritic cells in vitro. *Chemosphere* **177**, 56-64 (2017).

27. D. M. Hinton, J. J. Jessop, A. Arnold, R. H. Albert, F. A. Hines, Evaluation of immunotoxicity in a subchronic feeding study of triphenyl phosphate. *Toxicol Ind Health.* **3**, 71-89 (1987).

28. R. Fautz, H. Miltenburger, Influence of organophosphorus compounds on different cellular immune functions in vitro. *Toxicol In Vitro* **8**, 1027-1031 (1994).

29. D. Kharrazian, A. Vojdani, Correlation between antibodies to bisphenol A, its target enzyme protein disulfide isomerase and antibodies to neuron-specific antigens. *J Appl Toxicol.* **37**, 479-484 (2017).

30. L.-o. Chailurkit, W. Aekplakorn, B. Ongphiphadhanakul, The association of serum bisphenol A with thyroid autoimmunity. *Int J Env Res Public Health.* **13**, 1153 (2016).

31. S. Y. Lim, S. K. Ghosh, Autoreactive responses to environmental factors: 3. Mouse strain-specific differences in induction and regulation of anti-DNA antibody responses due to phthalate-isomers. *J Autoimmun.* **25**, 33-45 (2005).

32. S. Hirai *et al.*, Low-dose exposure to di-(2-ethylhexyl) phthalate (DEHP) increases susceptibility to testicular autoimmunity in mice. *Reprod Biol.* **15**, 163-171 (2015).

33. M. Kitaoka *et al.*, Effects on the local immunity in the testis by exposure to di-(2-ethylhexyl) phthalate (DEHP) in mice. *J Reprod Dev.* **59**, 485-490 (2013).

34. W. Qiu *et al.*, Toxic Effects of Bisphenol S Showing Immunomodulation in Fish Macrophages. *Environ Sci Technol.* **52**, 831-838 (2018).

35. A. J. Spanier, T. Fausnight, T. F. Camacho, J. M. Braun, The associations of triclosan and paraben exposure with allergen sensitization and wheeze in children. *Allergy Asthma Proc.* **35**, 475-481 (2014).

36. J. H. Savage, E. C. Matsui, R. A. Wood, C. A. Keet, Urinary levels of triclosan and parabens are associated with aeroallergen and food sensitization. *J Allergy Clin Immunol.* **130**, 453-460 (2012).

37. E. Maier *et al.*, Food preservatives sodium benzoate and propionic acid and colorant curcumin suppress Th1-type immune response in vitro. *Food Chem Toxicol.* **48**, 1950-1956 (2010).

38. M.-F. Lin, S.-Y. Shiau, Dietary l-ascorbic acid affects growth, nonspecific immune responses and disease resistance in juvenile grouper, Epinephelus malabaricus. *Aquaculture* **244**, 215-221 (2005).

39. A. Kim, B. H. Jung, P. Cadet, A novel pathway by which the environmental toxin 4-Nonylphenol may promote an inflammatory response in inflammatory bowel disease. *Med Sci Monit Basic Res.* **20**, 47-54 (2014).

40. C. Kim, P. Cadet, Environmental toxin 4-nonylphenol and autoimmune diseases: using DNA microarray to examine genetic markers of cytokine expression. *Arch Med Sci.* **6**, 321-327 (2010).

41. R. R. MacGregor, Alcohol and Immune Defense. *JAMA* **256**, 1474-1479 (1986).

42. M. J. Eckardt, T. C. Harford, C. T. Kaelber, Health Hazards Associated With Alcohol Consumption. *JAMA* **246**, 648-666 (1981).

43. J. NAGAYAMA, H. TSUJI, T. TAKASUGA, Comparison between brominated flame retardants and dioxins or organochlorine compounds in blood levels of Japanese adults. *Organohalogen Compd.* **48**, 27-30 (2000).

44. C. Thomsen, E. Lundanes, G. Becher, Brominated flame retardants in archived serum samples from Norway: a study on temporal trends and the role of age. *Environ Sci Technol.* **36**, 1414-1418 (2002).

45. Z. Xiao *et al.*, Determination of three brominated flame retardants in human serum using solid-phase extraction coupled with ultra-performance liquid chromatography-tandem mass spectrometry and gas chromatography-mass spectrometry. *Chin J Chromatogr (Se Pu).* **29**, 1165-1172 (2011).

46. M. Liu *et al.*, Metabolomic and transcriptomic analysis of MCF-7 cells exposed to 23 chemicals at human-relevant levels: estimation of individual chemical contribution to effects. *Environ Health Perspect.* **128**, 127008 (2020).

47. L. Peng *et al.*, Determination of organophosphate esters in human serum using gel permeation chromatograph and solid phase extraction coupled with gas chromatography-mass spectrometry. *Chin. J Anal Chem.* **43**, 1033-1039 (2015).

48. P. Li *et al.*, Concentrations of organophosphorus, polybromobenzene, and polybrominated diphenyl ether flame retardants in human serum, and relationships between concentrations and donor ages. *Chemosphere* **171**, 654-660 (2017).

49. F. Zhao, M. Chen, F. Gao, H. Shen, J. Hu, Organophosphorus flame retardants in pregnant women and their transfer to chorionic villi. *Environ Sci Technol.* **51**, 6489-6497 (2017).

50. S.-i. Kim, Y.-j. Yang, Y.-p. Hong, S.-C. Myung, S.-C. Kim, Distribution of serum bisphenol A diglycidyl ether and its metabolite in Korean adult men and its association with reproductive hormone levels. *Mol Cell Toxicol.* **11**, 71-78 (2015).

51. J. Sun, B. Chen, L. Zhang, D. Zhao, S. G. Li, Phthalate ester concentrations in blood serum, urine and endometrial tissues of Chinese endometriosis patients. *Int J Clin Exp Med.* **9**, 3808-3819 (2016).

52. A. M. Calafat *et al.*, Urinary concentrations of bisphenol A and 4-nonylphenol in a human reference population. *Environ Health Perspect.* **113**, 391-395 (2005).

53. C. Y. Lin *et al.*, Discovery of estrogen receptor alpha target genes and response elements in breast tumor cells. *Genome Biol*. **5**, 66 (2004).
